# Supplementary figures and images for: Bridging the gap between the evolutionary dynamics and the molecular mechanisms of meiosis: A model based exploration of the PRDM9 intra-genomic Red Queen
Source: PLoS Genet. 2024 May 20;20(5):e1011274. doi: 10.1371/journal.pgen.1011274 (PMC11142677; doi:10.1371/journal.pgen.1011274)

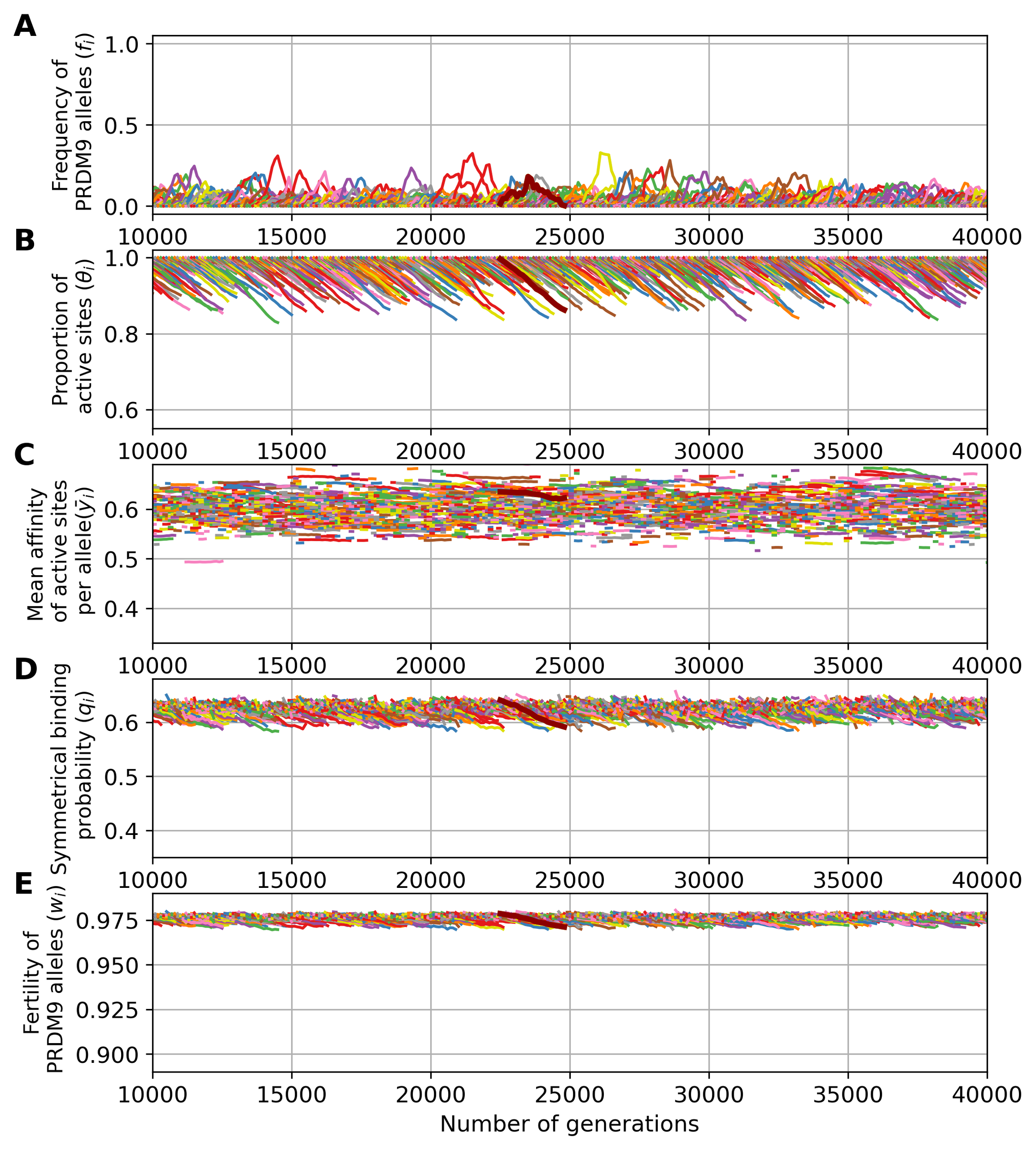

Supplement: S1 Fig — In all panels, each color corresponds to a different allele. Note that a given color can be reassigned to a new allele later in the simulation. Successive panels represent the variation through time of (A) the frequency of each PRDM9 allele and its corresponding (B) the proportion of active sites, (C) the mean affinity of active sites, (D) the probability of symmetrical binding and (E) the fertility. The thick line singles out the trajectory of a typical allele. (TIF) [file pgen.1011274.s001.tif]

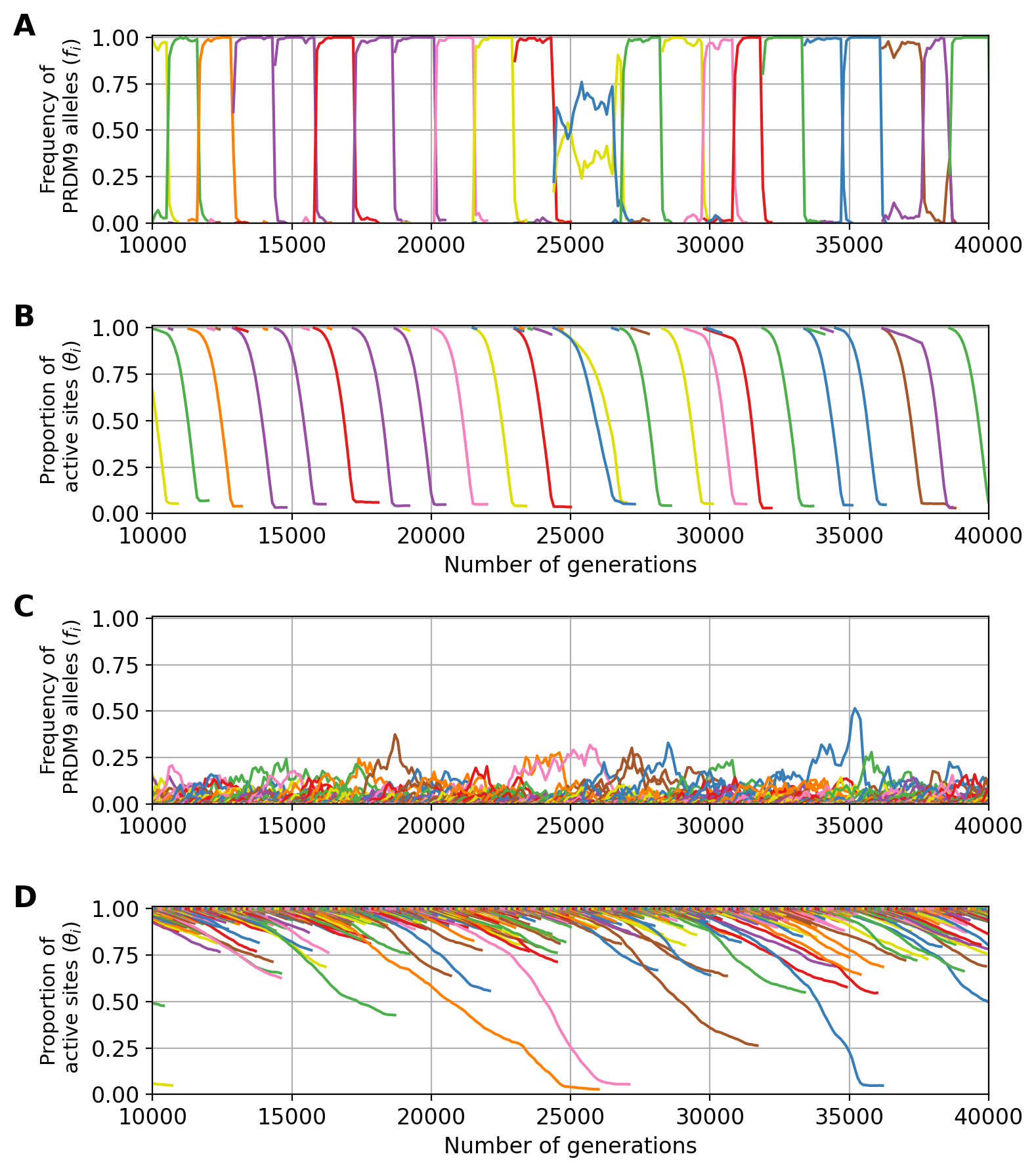

Supplement: S2 Fig — (A) and (B) correspond to the monomorphic regime (as in 3, u = 5 × 10^−6^, N = 5 × 10^3^ and v = 5 × 10^−5^), while (C) and (D) correspond to the polymorphic regime (as in 4, u = 5 × 10^−4^, N = 5 × 10^3^ and v = 5 × 10^−5^). In all panels, each color corresponds to a different allele. Note that a given color can be reassigned to a new allele later in the simulation. Successive panels represent the variation through time of (A) and (C) the frequency of each PRDM9 allele and (B) and (D) its corresponding proportion of active sites. (TIF) [file pgen.1011274.s002.tif]

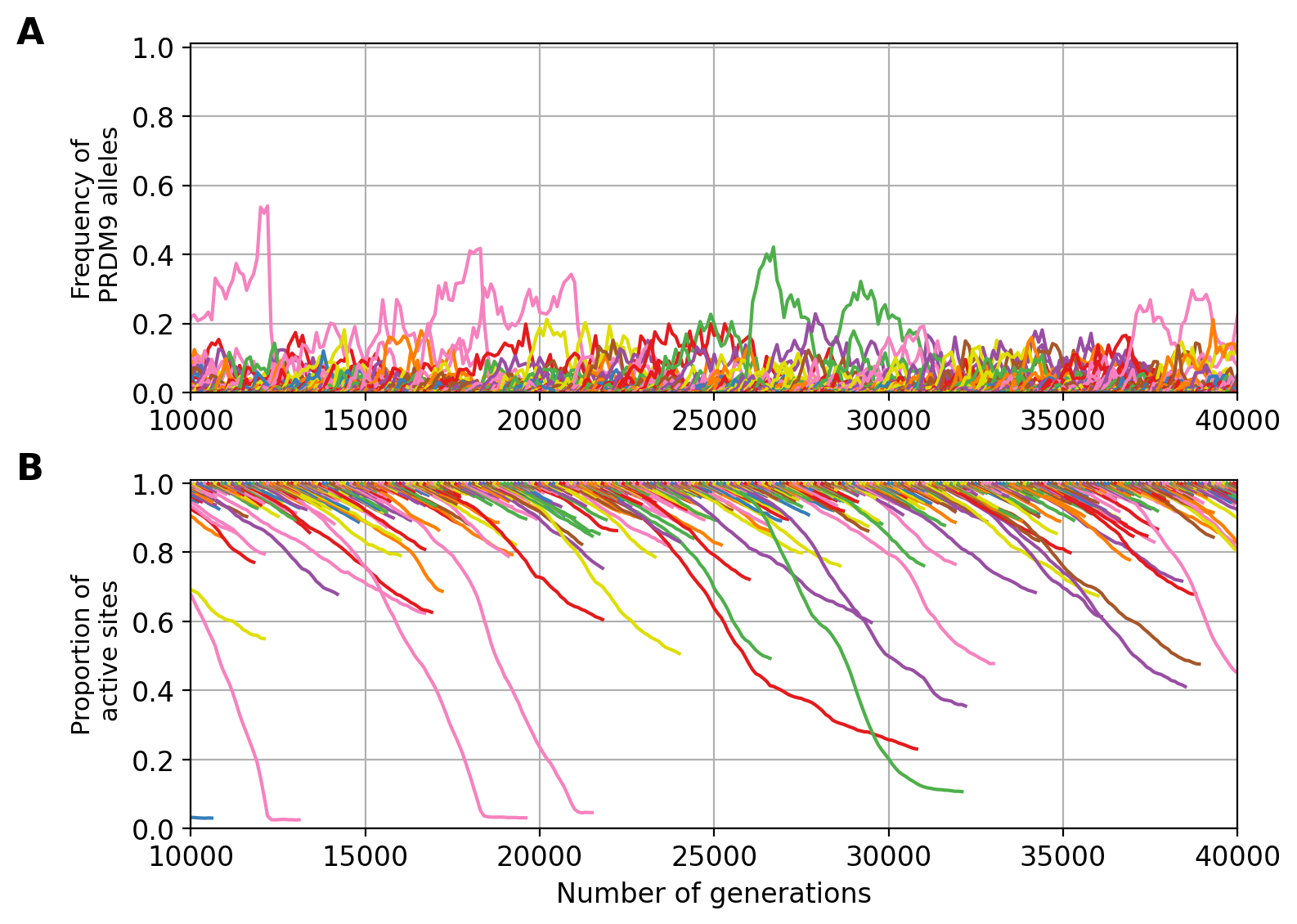

Supplement: S3 Fig — The simulation was run with u = 5 × 10^−4^, N = 5 × 10^3^ and v = 5 × 10^−5^. In all panels, each color corresponds to a different allele. Note that a given color can be reassigned to a new allele later in the simulation. Successive panels represent the variation through time of (A) the frequency of each PRDM9 allele and its corresponding (B) the proportion of active sites. (TIF) [file pgen.1011274.s003.tif]

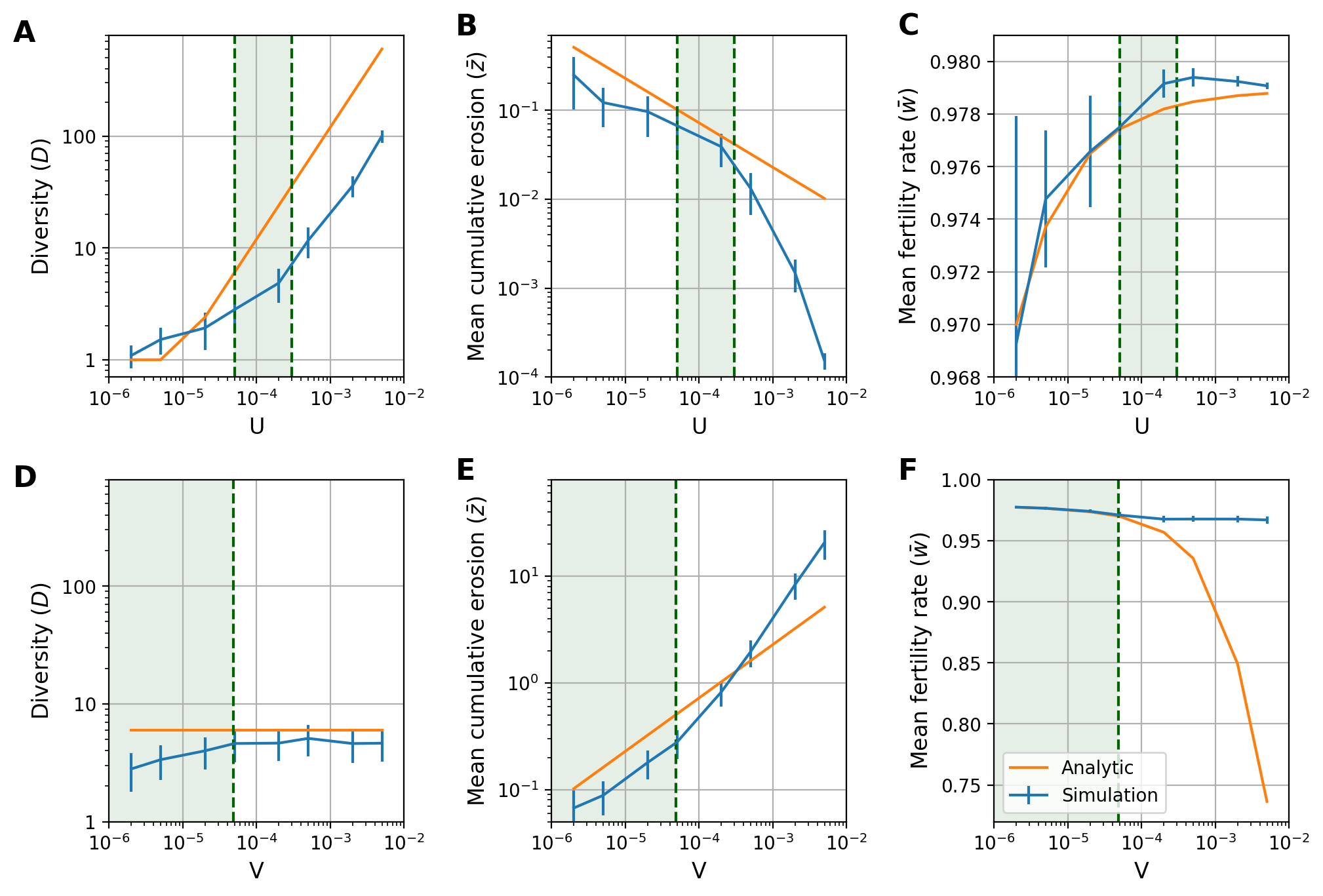

Supplement: S4 Fig — The statistics are: the PRDM9 diversity D as a function of u (A) and v (D); the mean erosion z¯ (i.e. the mean fraction of target sites that have been inactivated) at equilibrium as a function of u (B) and v (E); the mean fertility of the population w¯ as a function of u (C) and v (F). On each graph, the mean (blue line) and standard variation (blue vertical bars) over a simulation are displayed against the prediction of the analytical approximation (orange line). The area colored in green corresponds to the range of parameters for which the analytical model verifies the assumptions of a high diversity (1 < 4Nu < 100), a low erosion (z¯<0.5) and strong selection on new PRDM9 alleles (4Ns0 > 3). The analytical approximations presented here are plotted on panels A to F (orange curves), against the results obtained directly using the simulation program (blue curves). The model and the analytical approximation give qualitatively similar results in the range of parameters validating all the conditions (in practice, we consider that the analytical results should be valid in the following intervals for the model parameters: 1 < 4Nu < 100, z¯<0.5 and 4Ns0 > 3). Concerning PRDM9 diversity, substantial differences are observed between the simulation results and the analytical approximations, up to a factor of 10, in the scaling of u (panel A). However, the nature of the regime, polymorphic (many alleles segregating at the same time in the population) or monomorphic (only one allele present in the population at a time), is correctly predicted. In particular, we can say that the nature of the regime is directly and mostly determined by Nu and the level of erosion has almost no influence (panel D). Finally, the analytical approximations are less accurate for low and high u or high v. These correspond to strong erosion regimes (low u and high v) or to regimes with weak selection (high u), for which the assumption of the analytical developments are not met. (TIF) [file pgen.1011274.s004.tif]

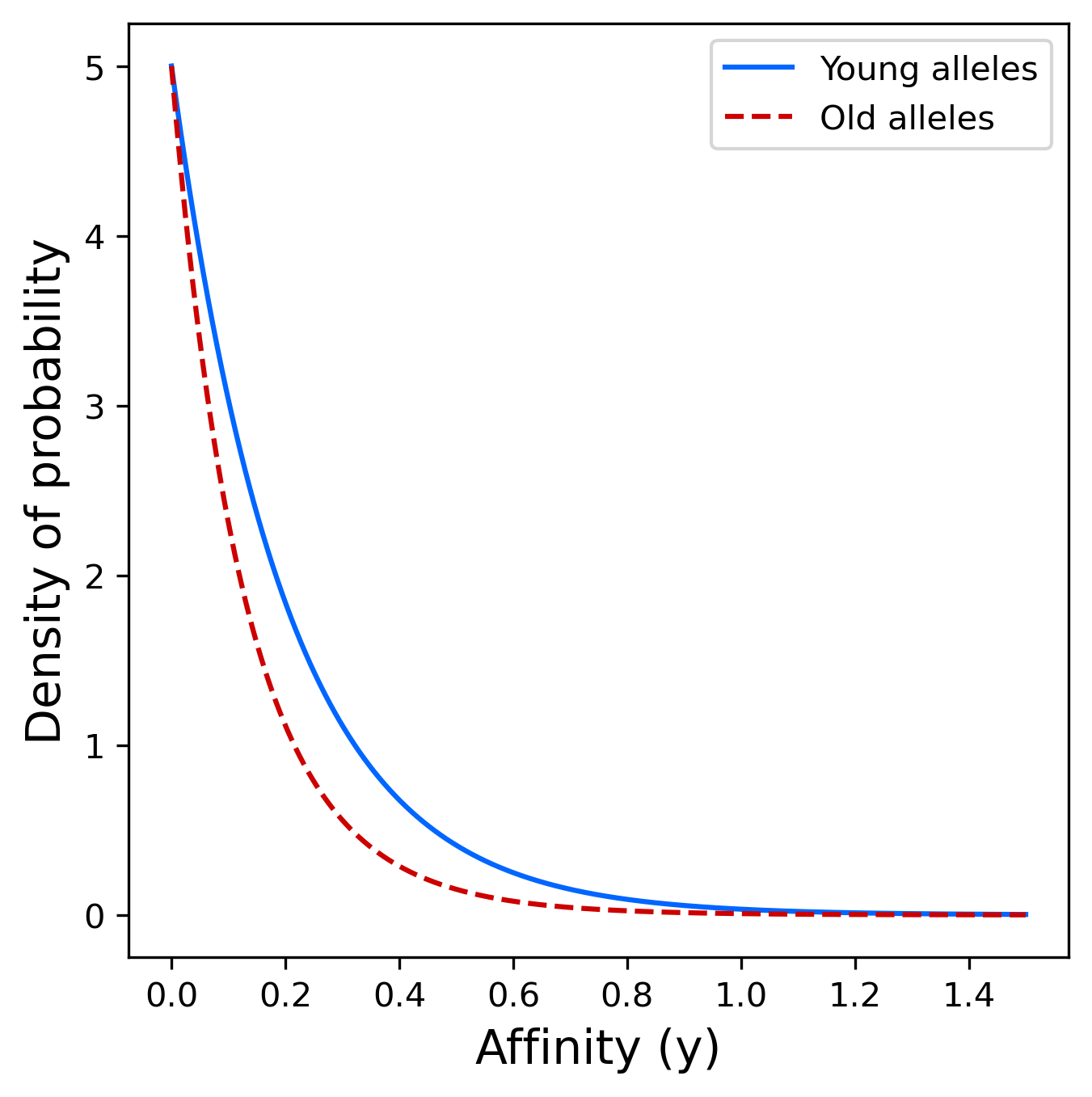

Supplement: S5 Fig — The continuous blue line corresponds to the affinity distribution for young alleles and the dotted red line corresponds to the affinity distribution for old alleles. (TIF) [file pgen.1011274.s005.tif]

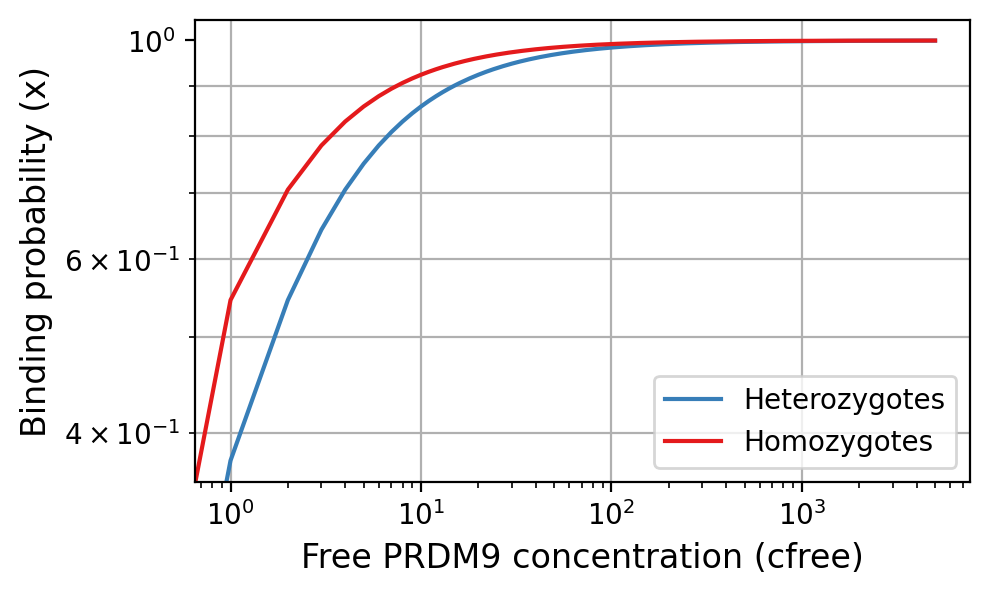

Supplement: S6 Fig — The binding probability for homozygotes is always higher than that for heterozygotes, but the difference between them decreases when the free PRDM9 concentration increases. (TIF) [file pgen.1011274.s006.tif]

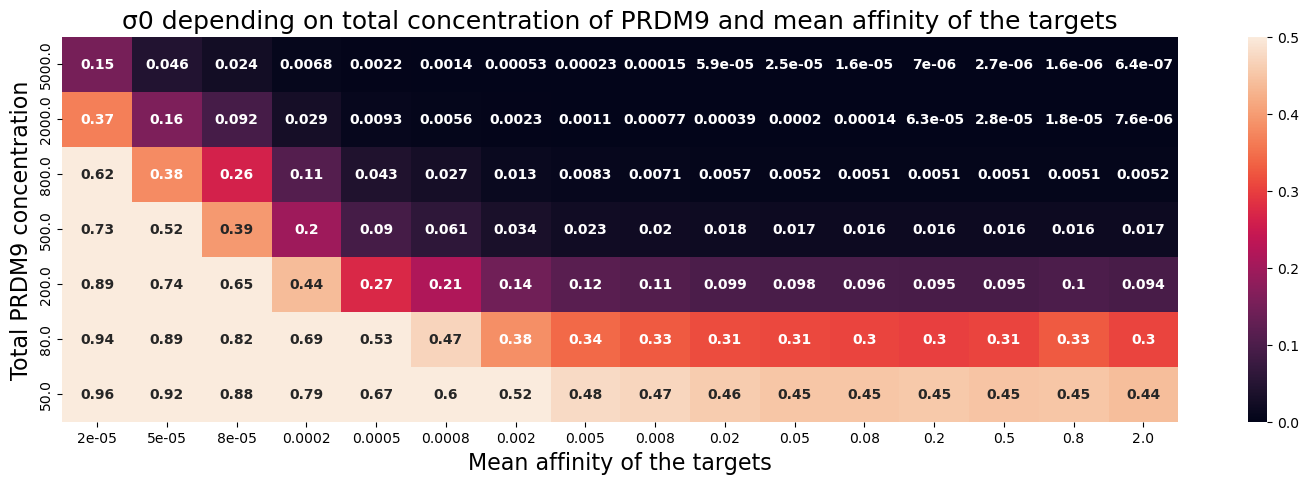

Supplement: S7 Fig — (TIF) [file pgen.1011274.s007.tif]
