# Supplementary material for: Bridging the gap between the evolutionary dynamics and the molecular mechanisms of meiosis: A model based exploration of the PRDM9 intra-genomic Red Queen
Source: PLoS Genet. 2024 May 20;20(5):e1011274. doi: 10.1371/journal.pgen.1011274 (PMC11142677; doi:10.1371/journal.pgen.1011274)
Supplement: S1 Text — (PDF) [file pgen.1011274.s009.pdf]

Bridging the gap between the evolutionary dynamics and the molecular mechanisms of meiosis : a model based exploration of the *PRDM9* intra-genomic Red Queen

Alice Genestier[1], Laurent Duret[1], Nicolas Lartillot[1,*]

1 Universite Claude Bernard Lyon 1, LBBE, UMR 5558, CNRS, VAS, Villeurbanne, France

* nicolas.lartillot@univ-lyon1.fr

Supplementary Text S1 : Empirical PRDM9 diversity in mouse subspecies

1 Introduction

Kono *et al.* (2014) genotyped the *Prdm9* ZF-encoding exon in 105 individuals, from 4 subspecies of mice (see Table S2 in [1]). In total, they identified 56 alleles encoding different ZF arrays. To analyze *Prdm9* diversity in natural populations, we focused on data from wild-captured mice (total=79 individuals). Kono *et al.* classified *Prdm9* alleles in 12 clusters (Ca, Cb, Cc, Cd, Ce, Da, Db, Dc, Dd, Ma, Mb, t). It should be noted that some of these alleles that belong to a same cluster share a large fraction of their target sites. For instance, *Prdm9* allele C3H (Da1 in [1]) is closely related to B6 (Da2), and these two alleles share ~30% of their targets ([2]). Similarly, PWD (Ma7) and MOL, which both belong to the Ma cluster share about 13% of their targets ([2]). Thus, the total *Prdm9* allelic diversity probably over-estimates the functional diversity (in terms of *Prdm9* target sites).
We therefore computed two estimates of *Prdm9* diversity:

- Dmax: diversity measured by considering all *Prdm9* alleles individually
- Dmin: diversity measured after grouping of *Prdm9* alleles per cluster

2 Summary

Species	NbIndividuals	NbAlleles	Heterozygous	NbClusters	Dmax	Dmin
M_m_molossinus	10	3	20.0%	2	1.80	1.60
M_m_domesticus	20	12	10.0%	4	6.30	2.24
M_m_castaneus	24	23	50.0%	7	12.52	5.82
M_m_musculus	25	27	48.0%	7	18.12	2.53

The number of individuals genotyped in *M. m. molossinus* is too small to estimate *Prdm9* diversity. In the three other subspecies, the total *Prdm9* diversity ranges from Dmax=6.3 to Dmax=18.12. If we consider the *Prdm9* diversity in terms of clusters of alleles, then diversity ranges from Dmin=2.24 to Dmin=5.82.

The true functional *Prdm9* diversity (in terms of target sites) is most probably between Dmin and Dmax.

References

[1] H. Kono et al. "Prdm9 Polymorphism Unveils Mouse Evolutionary Tracks". In: *DNA Research* 21.3 (2014), pp. 315–326. DOI: http://dx.doi.org/10.1093/dnares/dst059.

[2] F. Smagulova et al. "The evolutionary turnover of recombination hot spots contributes to speciation in mice." In: *Genes & Development* 30 (2016), pp. 226–280. DOI: http://dx.doi.org/10.1101/gad.270009.115.
