# Supplementary material for: Bridging the gap between the evolutionary dynamics and the molecular mechanisms of meiosis: A model based exploration of the PRDM9 intra-genomic Red Queen
Source: PLoS Genet. 2024 May 20;20(5):e1011274. doi: 10.1371/journal.pgen.1011274 (PMC11142677; doi:10.1371/journal.pgen.1011274)
Supplement: Attachment — Submitted filename: Meiosis review fpr PLOS Genetics.pdf [file pgen.1011274.s010.pdf]

What the authors have done:

The manuscript describes evaluation of a computer simulation model of the evolutionary processes acting on PRDM9-controlled meiotic recombination. This model follows PRML9 alleles and hotspot alleles in thousands of simulated individuals over many thousands of generations. PRDM9 alleles with new hotspot specificities arise by mutation. The presence of the new PRDM9 allele causes the 400 DNA sequences that, by chance, match its specificity to become hotspots. These hotspots are gradually lost as random mutation creates defective hotspot alleles that replace active ones by conversion. Now when a new PRDM9 allele arises by mutation it invades the population because its fertility is increased by the recombination it causes at its new hotspots. The paper emphasizes the significance of the newly discovered role of PRDM9 in directly promoting synapsis of homologous chromosomes, and incorporates this activity in its model. The authors used their model to investigate the effects of many aspects of PRDM9's action, including mutation rates of both PRDM9 and the hotspot sequences it acts at and PRDM9 dosage effects.

Assessment summary: This is an important piece of work. It has some scientific weaknesses that I think can be easily corrected, and it needs rewriting and figure changes to make the basic results accessible to researchers studying the molecular and cell biology of meiosis as well as to evolutionary theorists.

Major concerns about the science:

1. This work does not show the importance of PRDM9-mediated chromosome synapsis. This would require running 'control' simulations where PDRM9 plays no direct role in synapsis. See Line 186 comment below.

2. The initial simulations make a very unrealistic assumption that the concentration of a PRDM9 protein version in the cell does not depend on whether the cell is homozygous or heterozygous for that allele. This does not make biological sense, especially since different hotspots are assigned different affinities for PRDM9. Thinking about this is confounded because the initial model also assumed that PRDM9 was always in excess, making differences in the amounts of protein irrelevant.

   Later a more realistic 'Gene dosage' version of the model is introduced, where a meiotic cell homozygous for a particular PRDM9 allele contains twice as much of its version of the protein than a heterozygote does, making symmetrical binding twice as likely and thus dramatically increasing fertility. This dramatically changes the model's predictions. All the effort the reader has put into understanding the complex results in the first part of the paper turns out to have been wasted.

   Here's a sketch showing the differing assumptions:

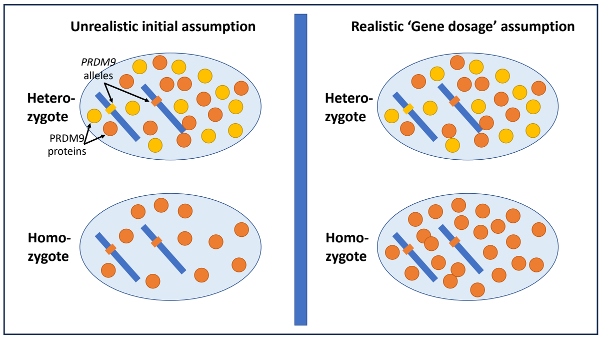

A general note about the writing: I think this article should try much harder to be accessible to both parties interested in meiosis: evolutionary biologists and molecular geneticists/cell biologists. At present it contains a lot of unexplained and poorly explained evolutionary biology jargon.

Many paragraphs are *much* too long, especially lines 153-185, 423-459 and 672-708. Any paragraphs more than 20 lines long should be re-evaluated.

The authors have been very careless in how they write the name of the *PRDM9* gene, of its alleles and of the PRDM9 protein. Between just the Title, the Abstract and the Author summary I found five different combinations of capitalization and italicization, apparently chosen randomly for each mention without regard to whether it was the gene, and allele or the protein. The authors should find out the correct forms and use them consistently.

Notes on specific points:

Abstract:

'Red Queen' is evolutionary genetics jargon that won't be understood by cell biologists and many geneticists. Either explain it at the very start (first describe what happens, and only then introduce the term), or don't use it at all in the Abstract. And minimize the use of it in the body of the manuscript too.

Spell out, early on, that we now know that 'hotspots' promote local crossing-over because they are high-affinity binding sites for PRDM9. This is not clear enough in the current Abstract.

'…hotspots are eroded…': The meaning of 'eroded' is not obvious here; describe what actually happens and explain that the term 'erosion' is used to describe this.

The Abstract ends with: "Finally, calibrating the model based on current empirical knowledge shows there is no need for much more realism in our model to correctly fit the empirical data." It's true that with appropriate choice of parameter values the model's predictions agree with what is seen in real meiosis and populations. But the model makes many simplifying assumptions, and relaxing any of these could dramatically change the outcomes.

Author Summary:

Be clear that meiotic recombination occurs in two ways, by random assortment of maternal and paternal homologs into gametes, and by crossing over. You may then, if you choose, say that since the model considers only a single chromosome you will use 'recombination' to mean 'crossing over' in this manuscript.

'instability of the recombination landscape': Most readers will not understand what this is referring to, so give a better explanation of the hotspot paradox.

Introduction:

Line

10 '..requires to articulate…' Maybe '… requires explicit descriptions of both…'

16 saying '…they cluster…' suggests that a single meiosis would have a cluster of closely spaced crossovers at each hotspot. Maybe say '…crossovers frequently occur at the same positions in independent meioses '.

21: Wait to introduce the 'zinc finger' until line 56.

36: The writing will be easier to follow if the terminology is consistent. Instead of 'replacement of 'hot' alleles by allelic motifs…' say 'replacement of 'hot' alleles by alleles...

38 The dBGC abbreviation is unnecessary; don't introduce it.

'mutations that weaken or inactivate hotspots.'

42 Again, I don't think 'erosion' is a very helpful term for this process. (It's used 80 times in the manuscript!)

48 It would be helpful to clarify here that the sequences recognized as hotspot sites by the new PRDM9 allele were already present by chance in the genome.

56 Does the reader need to know that the zinc finger is encoded by a mini-satellite? Why is this significant?

62 'Wright-Fisher models'. OK, from here on I'm going to stop suggesting ways to make the manuscript intelligible to meiosis researchers who aren't evolutionary biologists.

68 'induced by PRDM9' is ambiguous. How about 'seen in PRDM9 heterozygotes with incompatible PRDM9 alleles'?

75 How about 'In a F1 hybrid, each of the two PRDM9 alleles has eroded its targets in the genome of its parental strain, but not in the other other strain's genome.'?

77 (They're not really in trans or in cis, since the PRDM9 allele is only on the homologs of one chromosome.) How about 'Each PRDM9 allele will therefore tend to bind preferentially to the still active hotspot sites present on the chromosomes inherited from the other parent, but not to the homologous but eroded sites on the chromosome from its own parent.'?

93 Do DSBs often get repaired by pairing with a strand in the sister chromatid? If not I'l leave this out.

96 If the homologs fail to synapse the meiosis will be aborted.

103 Huh?

107-109 Maybe, instead of saying 'panmictic', say 'within a single population' – this better reinforces the contrast with hybrids formed between members of two separately evolving populations. A diagram would be helpful.

Results

126 Don't just dump the reader in at the deep end with the model. The Results should start with an overview that briefly describes the components and events of the model and explains how these approximate the components and events of meiosis in a real population. Since the Methods will be placed at the end of the paper, the authors shouldn't assume that the reader has already read them.

Skipping over to the Methods section:

Is this description correct? (I'm not expecting this to be used to introduce the model – I just want to try to lay out my understanding.)

1. The model simulates one chromosome (2 homologs per individual) in 5000 individuals of a randomly mating population.
2. Each chromosome has one PRDM9 locus.
3. Each chromosome has 400 loci that are binding sites for its PRDM9 protein (potential hotspots).
4. These binding sites have differing affinities for PRDM9 (some are stronger hotspots than others)
5. The relative positions of these hotspot loci on the chromosome are not specified.
6. The population is followed over many generations.
7. Each generation consists of mutation, production of gametes by meiosis and then random fusing of gametes from pairs of individuals to create the next generation of individuals.
8. When a PRDM9 locus mutates its protein acquires a different hotspot-binding specificity. A new array of 400 hotspot loci is then created for each new PRDM9; these simulate pre-existing DNA sequences that now will act as hotspots for the new PRDM9 protein.
9. When a hotspot allele mutates it becomes unable to bind its cognate PRDM9 protein (it becomes a cold spot).

622 Fig. 9 has several problems, some more serious than others:

1. Why do the drawings show the hotspots for PRDM9 alleles that the individual does not have? In Fig. 9A, the first and fourth individuals are homozygous at PDRM9 and thus should have only one type of hotspot (yellow and green respectively). In Fig. 9B, only the third individual has a dark-red PRDM9 allele, so why are the hotspots for this allele shown in all the individuals? And why show the yellow hotspot alleles in this individual, when its only PRDM9 alleles are green and dark red? Similarly, there should be no dark-red hotspots in panels C, D and E. Removing the irrelevant target sites from the drawings will make the representation both simpler and more accurate.
2. The representation of and spacing between sister chromatids in panels B, C, D and E is exactly the same as of homologs in Panel A reinforcing the all-too-common confusion between sisters and homologs. At least draw the sisters as being closer together than the homologs were in Panel A.
3. The Panel B individual whose meiosis is shown in Panels C, D and E should be indicated by a purple rectangle, not a black oval, since Panels C, D and E are enclosed in a purple rectangle.
4. In Panels C and D, binding of PDRM9 to its cognate hotspots should be indicated by filled circles of the appropriate color representing PDRM9 protein (like those representing its gene), not by skinny arrows.
5. Since Panel D is supposed to represent synapsis of the homologs, put them closer together, at least at the symmetrical site.
6. Are the fat yellow arrows inside the blue rectangle in Panel D supposed to indicate that the homologs are being brought together at the site of the symmetrical binding? It should be possible to find a clearer way to represent this. And what does the skinny-line rectangle below indicate? It has symmetrical binding of PDRM9 but no fat arrows.
7. The red rectangle in Panel E indicates gene conversion at the top green hotspot
8. Shouldn't Panel E show the result of the crossover? Instead it shows the boxed chromatid arms still in their original positions.
9. Panel F is an orphan – I completely overlooked it because it's so far over to the right.
10. Don't use similar rectangles to represent different kinds of events and relationships (individual, crossovers, potential crossovers, synapsis, gene conversion).

Continuing through the Methods:

629 Maybe have a little plot somewhere showing the distribution of target site affinities at their creation (because I'm not sure what 'an exponential law of parameter $\bar{y}$' looks like) and the distribution at the time their cognate PRDM9 allele goes extinct?

639 The two alleles of each new hotspot site are assigned identical affinities. This is reasonable, but with no mutations affecting hotspot affinity, the model can't include conversion of a strong hotspot to a weak one, likely a very important component of hotspot erosion.

645 This is reasonable since hotspot alleles newly arisen by mutation would be at a very high risk of being lost by conversion.

663 There are 400 target loci (hotspot positions), but each is present on four chromatids in the meiotic cell. Is the binding calculation done for each site on each chromatid in turn?

664-672 I do not understand what this analysis is accomplishing.

683 'which **the model assumes** are essential for crossing-over' ?

686: 'on which to hybridize' - replace with 'to base pair with'?

697 'The chromosomal segments on either side of the chosen site are exchanged' Does 'on either side' mean 'on both sides'? That would not be recombination at all. Should it be 'on one side'?

702: The hotspot allele at the site of the DSB that became the crossover also gets converted, right? Oh, this is irrelevant if non-dead hotspot alleles are always identical (see line 639 comment above).

705 …to become a gamete'.

787 'the number of bound sites per allele was set to h = 400'??? What does 'bound sites per allele' mean? I would expect something like 'sites recognized as hotspots by each PDRM9 allele's protein'.

Back to the start of the Results:

126 and 128 'Intragenomic Red Queen' and 'Wright Fisher simulation' will only be intelligible to population geneticists.

135 The appropriate response on seeing Fig. 1 is "YEOW!!" If you move the figure until after the explanation in the next paragraph it won't be nearly so daunting.

Legend to Fig. 1

'In all panels, different colors correspond to different PDRM9 alleles that have newly arisen by mutation.'

Replace 'Successive panels' with 'Each panel'.

Many events are crowded together in each panel, making it difficult to align events in different panels. Adding faint vertical lines every 5000 generations would help.

139 The paper will be more accessible to cell and molecular biologists if technical terms such as 'monomorphic regime' are only introduced when really needed.

140 It would be helpful to have a figure explaining the events in the life of a single PRDM9 allele first. It would be most useful if this was a run with a lower μ, so the 'old' allele hangs around longer. See suggestion at line 200 below.

How often do we expect a new PRDM9 allele to arise, with μ=5x10^{-6}? N=5000, 2 alleles each, so about one every 500 generations? Yes, that's about what we see.

The new PRDM9 allele invades because it initially has more active hotspots than the resident allele(s) and thus more successful meioses. But inactive hotspots start to accumulate, initially due to mutation and then also due to conversion of active hotspots. This reduces the meiosis advantage, initially very slowly by mutation and then faster and faster as the probability of conversion rises. But the previous PRDM9 allele can't come back since loss of hotspots is irreversible (though check out the purple pone that arises at generation 17,000). So the frequency of the new allele continues to increase until it is fixed or until a new allele arises by mutation. The longer it has persisted the fewer active hotspots remain and the faster it is replaced once a new allele arises.

Usually, fertility is a property of an organism, not an allele. Is the fertility plotted in Fig. 1E the mean fertility (probability of successful meiosis) of the diploid individuals carrying this allele? Or, as lines 192-3 imply, of all the individuals in the population, regardless of which PRDM9 allele(s) they are carrying? But that can't be right, because the plots show allele-specific fertilities (and allele-specific symmetrical binding probabilities). This certainly needs clarification.

Why does the proportion of active sites rarely fall below 0.6? Are they just not shown once their cognate PRDM90 allele is lost? Even though the old allele still has lots of reasonably active hotspots, it can't produce as many gametes as the new allele and so gradually goes extinct.???

141 A reader who has not carefully read the Methods (they're at the end of the paper) won't know what 'activity' means here. It's not a property of each PRDM9 allele, but a biochemical measure of how efficiently a PRDM9 protein can cause crossovers given its current supply of hotspots, right?

143 replaced by a newly arisen PRDM9 allele that recognizes a different hotspot sequence motif.

165 'bounding sites'???

The terminology 'activity' and 'active sites' and 'affinity' creates confusion: 'Activity' is a property of a PRDM9 allele -they all start out with the same activity, but this changes as the allele's target sites change. But being 'active' is a property of a target site (hotspot) – sites start out active and become inactive due to mutation or conversion. Only 'active' sites have 'affinity'; that's a stable property of a given site, indicating how efficiently they bind to PRDM9 protein.

166-168 The relationship between these two sentences is unclear. Maybe just combine them into one statement.

169 Delete '…are eroded more rapidly. This last point is also expected, since the sites of high affinity…' and '(the binding probability = cy 170 1+cy is higher when the affinity y is higher)'.

180 of the affinity of the hotspots for the PRDM9 protein?

181 'all sites of a given allele' is confusing. Instead 'all target sites of a given PRDM9 allele'?

186-189 Well no, the importance of symmetrical binding hasn't been established in the model. How does the model outcome change if the requirement for symmetrical binding is removed? For example, synapsis (and successful meiosis) could instead be made to depend on the number of DSBs in the chromosome.

192-195 Symmetrical binding probability and fertility 'are defined, for each allele, as a mean over all diploid genotypes segregating in the population at any given time.' This definition makes panels D and E quite misleading. I don't understand how the plots can show what appear to be allele-specific symmetrical binding probabilities and fertilities if this is the case. For example, around generation 16,000 we see a pale green line and a turquoise line, which would seem to imply that the data are specific to the segregating pale green and turquoise PRDM9 alleles.

Why does being heterozygous for 'old' and 'new' PDRM9 alleles (with different specificities) increase the likelihood of symmetrical binding? Is frequent symmetrical binding by two 'new' PRDM9 proteins just compensating for the increasingly rare symmetrical binding by two 'old' PDRM9 proteins?

Is it just that when the individual is heterozygous there's only half as much of each type of PRDM9 protein? No, that would explain why the probability of symmetrical binding by the new allele goes up as it becomes more common, but not why the probability of symmetrical binding by the old allele goes up as the new one becomes common.

This paper ***really*** would benefit from a one-cycle figure before the current Fig. 1.

Usually, fertility is a property of an organism, not an allele. Is the fertility plotted in Fig. 1E the mean fertility (probability of successful meiosis) of the diploid individuals carrying this allele? Or, as lines 192-3 imply, of all the individuals in the population, regardless of which PRDM9 allele(s) they are carrying? But that can't be right, because the plots show allele-specific fertilities (and allele-specific symmetrical binding probabilities). This certainly needs clarification.

200 Specifically, this mutation rate is ***100 times higher*** (5×10^{-4}) and very unrealistic, since such a mutation rate would generate very many nonfunctional PRDM9 alleles and lead to extinction of the whole population. It would be much more informative to instead show results with a narrower range of μ, say 2×10^{-6} (for an initial explanatory figure), 5×10^{-6} for what is now Fig. 1 and 2×10^{-5} for what is now Fig. 2.

Does the model incorporate any cost to the population of the declining fertility due to hotspot erosion, under either the low or high mutation rate scenarios? If this was in place, and the PRDM9 mutations were modeled

to include a reasonable proportion of non-functional recessive alleles, the authors could test a range of mutation rates to see if there is an optimum.

209 Perhaps provide a supplementary figure where the panels of Fig. 1 and Fig. 2 are drawn to the same scales.

236 Doesn't this assume that only newly-mutated targets participate in conversion?

There's a big jump in line numbers here because I found the 'scaling' analysis (lines 211-330) hard to follow.

332 In heterozygotes, half of the PRDM9 protein in the cell is from one allele and half is from the other. In the previous version of the model a heterozygote has twice as much total PRDM9 protein as a homozygote, a situation that does not make sense biologically. In the new version of the model the total amount of PRDM9 protein in an individual is the same for heterozygotes and homozygotes.

345-365 This is a striking result. During the extended regime with a stable high-frequency PRDM9 allele, the proportion of active sites decays to about 0.2 and then stabilizes. Why does it stabilize? And is the bulk of the meiotic recombination being done by the low-frequency alleles? -If the model included population fitness would the population just die out?

436 The hotspot mutation rate used in the simulations was 500-fold higher,

437 Explain here (or better, earlier) why the real mutation rate is so high (not point mutations but recombination and indels in the Zn finger). Discuss the nature of the real mutations typically seen and their effect on binding ability and binding specificity.

450 'one meiosis per individual' means one meiosis attempt per individual each time it is chosen to attempt meiosis. However, an individual may be chosen several times in one generation. In the new alternative, the chosen individual is allowed several attempts to produce a functional gamete.

472 Tell the reader about the typical natural diversity and heterozygosity for PRDM9 alleles earlier (in the Introduction).

The model assumes that new PRDM9 alleles have specificities that don't overlap with the parental allele's specificity,

506 "Altogether, we deduce that there is no need for much more realism in our model to correctly fit the empirical data." This is a dangerous statement. The model may make unrealistic assumptions about factors that are critical for determining its outcome, and still produce output that matches available data. For example, the current model assumes that every generation produces enough successful meiosis to create the same number of individuals in the next generation. Relaxing this assumption might show that, when a PRDM9 allele persists for a long time, the population goes extinct because its fertility falls below a critical level.

Discussion

524 No, I don't think the need for the second function of PRDM9 has been shown, because versions that did not have this feature were not tested.

535-536 'hybrid context' and 'panmictic context'??? Again, the word 'panmictic isn't helpful. Maybe again spell out the situations a bit.

545 This description of the 'gene dosage' version of the model seems to match what I wrote above (line 332) trying to describe the original version of the model. Since this is the only arrangement that makes biological sense, I don't understand why all the initial modelling (e.g. Figs 1 and 2) would have been done using a different version. Why waste the reader's attention on a model version that should be discarded?

547 This is because the initial assumption that PRDM9 was not limiting has been removed, right? Baker 2022 cites refs saying hotspots do compete for PRDM9.

623 In Table 2 μ is listed as both a parameter and a variable, with slightly different definitions.

Linkage is not discussed (a search didn't find the word). Since the model considers only a single chromosome, and the positions of the PRDM9 locus and the hotspots are not specified, in the Discussion the authors should consider the possible effects of linkage between hotspots and between the PRDM locus and the hotspots it acts at.

Since the hotspots matching each new PRDM9 allele were initially at mutational equilibrium on their genome, new ones are being created by mutation at the same rate that existing ones are destroyed by mutation. It's reasonable for the model to neglect the creation of new ones (they're initially heterozygous so destroyed by gene conversion the first time they're used), but the authors should explain this.
