# Supplementary material for: Bridging the gap between the evolutionary dynamics and the molecular mechanisms of meiosis: A model based exploration of the PRDM9 intra-genomic Red Queen
Source: PLoS Genet. 2024 May 20;20(5):e1011274. doi: 10.1371/journal.pgen.1011274 (PMC11142677; doi:10.1371/journal.pgen.1011274)
Supplement: Attachment — Submitted filename: GENESTIER_et_al_2023_Response_to_Reviewers.pdf [file pgen.1011274.s011.pdf]

Dear Dr Genestier,

Thank you very much for submitting your Research Article entitled 'Bridging the gap between the evolutionary dynamics and the molecular mechanisms of meiosis: a model based exploration of the PRDM9 intra-genomic Red Queen' to PLOS Genetics.

The manuscript was fully evaluated at the editorial level and by independent peer reviewers. All the reviewers appreciate the modelling approach to understand the behaviour and evolution of PRDM9, and find the new insights this results in interesting - particularly the impact of dosage effects, interacting with fitness, on the evolutionary dynamics of the protein and hence the recombination landscape.

However, the reviewers also raised some substantial concerns about the current manuscript. Based on the reviews, we will not be able to accept this version of the manuscript, but we would be willing to review a much-revised version. We cannot, of course, promise publication at that time.

Should you decide to revise the manuscript for further consideration here, your revisions should address the specific points made by each reviewer. We will also require a detailed list of your responses to the review comments and a description of the changes you have made in the manuscript. In particular, please carefully address the following points raised in the review process:

1) Concepts. All of the reviewers noted that several concepts should be defined/described to enable them to be fully accessible for non-specialist readers. In particular, please ensure the manuscript includes clear definitions of (i) the "erosion" of hotspots (which I think here means their loss via the action of the hotspot paradox), and (ii) the Red Queen hypothesis (in the general sense), used throughout this work. It would also be helpful to (iii) explain that in the case of interest, hotspots are positioned due to their being strong binding sites of PRDM9, (iv) explain the significance of the minisatellite nature of the PRDM9 ZF array, in allowing the potential for the rapid accumulation of new, functional alleles with completely different hotspots, and for a high mutation rate. (v) Explain the "Wright-Fisher" model (briefly). (vi) make sure "symmetrical binding" is well defined.

We have reformulated many of those ideas in the introduction, so as to define those concepts more clearly. In particular:

(i) erosion: 'This self-destruction phenomenon, commonly called the "hotspot conversion paradox" (Boulton et al 1997), leads to the progressive inactivation, hereafter called erosion, of PRDM9 binding sites at the genome scale..'

(ii) Red Queen: as suggested by Reviewer 2, we now first explain the model and then say that it was suggested to call it a Red Queen (while explaining the reason for this suggested terminology).

(iii) hotspots: we have added: 'The hotspots therefore correspond to strong binding sites of the PRDM9 protein.'

(iv) connection between the minisatellite structure and the high mutation rate of the exon encoding the PRDM9 binding domain: 'Second, the zinc finger domain is encoded by a mini-satellite, which mutates rapidly (Jeffreys et al 2013) by a combination of point mutations and unequal crossing over between the sequence repeats. This allows for the rapid accumulation of new combinations of zinc fingers, and thus of new alleles recognizing different hotspots,... '

(v) We have realized that the 'Wright-Fisher' terminology does not refer to anything specific, apart from a general model of a reproducing population with non-overlapping generations. So, we have removed this term in the introduction and results section.

(vi) asymmetrical versus symmetrical binding: 'The asymmetry, here corresponds to the binding of one PRDM9 protein at a specific site on one chromosome but not on the homologue at the same position.'. Later in the introduction:' in addition to being responsible for the recruitment of the DSB machinery, it would also facilitate the pairing between the homologous chromosomes, thanks to its symmetrical binding (i.e. simultaneous PRDM9 binding to the same target site on both homologues).'

2) Dominance of PRDM9 alleles (reviewer 1). Please explain if this is accounted for in the model and/or the implications of dominance of some PRDM9 alleles which can be strong in nature, and might of course evolve for model-based predictions of PRDM9 evolution. [NB dominance does not appear to be a purely emergent phenomenon from erosion, because in several studies it is observed even for pairs of alleles whose binding sites have not been eroded in the genome of interest. For example, does the existence of dominance provide a selective advantage to more dominant newly-arising alleles.]

In our model, all the alleles have almost the same mean affinity for their targets. To be accurate, due to the random drawing of the affinity of each binding site of a new allele, there is in fact some variance in dominance effect, but this is weak – in any case, we did not intentionally try to model intrinsic dominance by those means. We now discuss this simplifying assumption of our model at the very end of the discussion.

3) Resolution of double strand breaks (DSBs) – please explain the implications of repair via crossovers, non-crossovers (which contribute to motif erosion) or sister chromatid repair (which likely doesn't) to the model, and add to the discussion the possibility that at heterozygous hotspot loci the proportion of DSBs repairing using the sister chromatid might be higher.

In the model presented in this manuscript, we do not allow the repair with the sister chromatid by simplification. If we were to take this into account, this would mostly slow down the process of erosion. In any case, we now mention the existence of sister chromatid repair in the introduction, citing Li et al. We make it clear later in the text that the model assumes that all DSBs are repaired with the homologue. Finally, we discuss the consequences of this (at the end of the discussion).

4) Nomenclature, which the reviewers note is not consistent/correct in various places: several examples are in the abstract. In particular, please check/use standard nomenclature for e.g. gene names: use upper case non-italicised letters to refer to a protein, and italicised letters to refer to the gene/DNA sequence, capitalising the whole name for human gene names or only the first letter for the mouse gene, and make it clear to the reader whether the gene (natural e.g. if considering alleles or mutations to alleles)/protein (natural e.g. if considering binding properties, etc etc.) is being referred to.

*We have clarified the nomenclature: using 'PRDM9' (italicized) for the gene and '*PRDM9' *for the protein product.*

5) All the reviewers highlighted typos that should be fixed, suggest removing unnecessary abbreviations (e.g. "dBGC"), and also suggest that paragraphs might be shortened in a number of places.

We have simplified the terminology (in particular, we have removed 'dBGC', or 'activity', which were indeed not so useful). For those terms that we deem are important (monomorphic / polymorphic, erosion, Red Queen), we have tried to clearly define them upon first using them. The only exception to that rule may be the abstract, where we still mention the word Red Queen (because this has been the terminology since the first time the model was proposed – in fact, even in the title of the original article of Ubeda and Wilkins).

We have tried to make the text globally more concise in several places where our original text was perhaps too long-winded and redundant. We have also split the longest paragraphs to ease reading. As a way to compensate for the additional paragraphs that were added to bring the requested clarifications, we propose to push the little section presenting the

bi-dimensional scaling experiments with and without dosage, along with the two figures that were associated to it (former Figures 6 and 7) in the Supplementary Material, since this part was quite technical, and was mostly there as a check of our mathematical criterion for predicting when dosage would drastically reduce the genetic diversity of PRDM9. We now just refer to those checks very briefly in the main text.

6) Please address the other points made by individual reviewers in full, including in particular suggestions for figure modifications by reviewers 2 and 3, and the suggestion of reviewer 1 to discuss implications of potential migration/population structure for the models.

Concerning the figures (and tables):

- we have added a little figure in the introduction (new Figure 1) to explain the idea about the role of symmetrical binding in facilitating chromosome pairing.

- we have added a paragraph at the beginning of the Results section explaining the model. We have also moved the explanatory figure, which was originally in the method, up to this point of the manuscript (as the new Figure 2).

Concerning population structure and migration: we now briefly mention this point at the very end of the discussion, as one potential factor that could contribute to maintaining a higher diversity at the PRDM9 locus, compared to what our model predicts.

7) Reviewer 2 describes a relaxation of the assumption that PRDM9 dosage is not limiting as being a component of the model incorporating dosage, and this seems a logical description of the later model. In the discussion, though, this is highlighted as a difference with the Baker et al. approach: "in our model, the amount of PRDM9 protein is not limiting", going on to say that the model of this manuscript does not include competition between PRDM9 targets. Can you either reword or add an explanation of this fairly subtle difference, whereby gene dosage can have an impact (suggesting more PRDM9 corresponds to more binding) but without there being actual competition between binding sites? Further, please specify in what sense can this correspond to gene dosage being "non-limiting", if higher dosage of an allele means higher binding to sites and higher fitness?

This is indeed a fairly subtle point, which we now explain in more detail, in particular in the subsection of the discussion about the comparison with the theoretical work of Baker et al, where it matters most.

8) The manuscript contains the line “this step of meiosis is critical, since it is what will allow the two homologues to associate, realize a CO and form their synapsis”. Perhaps reorder to place synapsis in front of CO, because at present this reads as though CO is required for or precedes synapsis, but this is not the case - rather COs are linked most closely to (avoiding) aneuploidy. DSBs are likely required for synapsis in the species of interest here; it is further very likely that homologue engagement (which may involve either CO or NCO events) is also essential. There are a few other places where this potential misunderstanding also occurs, e.g. line 174. (Available evidence from the Forejt lab’s papers also suggest ~20Mb of homozygosity, enough for ~1 DSB to reliably occur, but not for a CO, is enough to restore synapsis of a chromosome.)

These two sentences have been removed altogether (as a result of our attempt at making the manuscript globally shorter).

If you decide to revise the manuscript for further consideration at PLOS Genetics, please aim to resubmit within the next 60 days, unless it will take extra time to address the concerns of the reviewers, in which case we would appreciate an expected resubmission date by email to plosgenetics@plos.org.

If present, accompanying reviewer attachments are included with this email; please notify the journal office if any appear to be missing. They will also be available for download from the link below. You can use this link to log into the system when you are ready to submit a revised version, having first consulted our Submission Checklist.

To enhance the reproducibility of your results, we recommend that you deposit your laboratory protocols in protocols.io, where a protocol can be assigned its own identifier (DOI) such that it can be cited independently in the future. Additionally, PLOS ONE offers an option to publish peer-reviewed clinical study protocols. Read more information on sharing protocols at https://plos.org/protocols?utm_medium=editorial-email&utm_source=authorletters&utm_campaign=protocols

Please be aware that our data availability policy requires that all numerical data underlying graphs or summary statistics are included with the submission, and you will need to provide this upon resubmission if not already present. In addition, we do not permit the inclusion of phrases such as "data not shown" or "unpublished results" in manuscripts. All points should be backed up by data provided with the submission.

While revising your submission, please upload your figure files to the Preflight Analysis and Conversion Engine (PACE) digital diagnostic tool. PACE helps ensure that figures meet PLOS requirements. To use PACE, you must first register as a user. Then, login and navigate to the UPLOAD tab, where you will find detailed instructions on how to use the tool. If you encounter any issues or have any questions when using PACE, please email us at figures@plos.org.

PLOS has incorporated Similarity Check, powered by iThenticate, into its journal-wide submission system in order to screen submitted content for originality before publication. Each PLOS journal undertakes screening on a proportion of submitted articles. You will be contacted if needed following the screening process.

To resubmit, use the link below and 'Revise Submission' in the 'Submissions Needing Revision' folder.

We are sorry that we cannot be more positive about your manuscript at this stage. Please do not hesitate to contact us if you have any concerns or questions.

Yours sincerely,

Simon Myers
Guest Editor
PLOS Genetics

Gregory P. Copenhaver
Editor-in-Chief
PLOS Genetics

Reviewer's Responses to Questions

Comments to the Authors:
Please note here if the review is uploaded as an attachment.

Reviewer #1: Genestier, Duret, and Lartillot present a statistical and theoretical treatment of the possible mechanisms underlying the rapid evolution of the recombination hotspot protein PRDM9. Specifically, they created a mechanistic simulation that relates the effects of PRDM9 binding symmetry, which has been shown to affect synapsis and fertility, to the

erosion of its binding sites and the rise of new PRDM9 alleles. They also created analytical approximations to these simulated results and explored parameter regimes that recapitulate empirical observations. In doing so, they could conceptually bridge recent empirical observations about the molecular behaviour of PRDM9 in meiosis to its long-term evolution. This is a substantial update to previous models that only accounted for the mutation of PRDM9 and erosion of its hotspots, without accounting for the importance of binding symmetry. This represents an important contribution, which can generate new testable hypotheses about the molecular behaviours of PRDM9, and it helps to illuminate why PRDM9 is observed to be both highly polymorphic yet often has few high-frequency alleles or allele groups. I enthusiastically recommend it for publication, and I only have minor suggestions that may improve the manuscript:

1) Overall: I imagine these will naturally be corrected in the editorial process, but there are a large number of typos, and the text could afford to be shortened considerably to highlight the most important results.

We have done our best to fix all of the typos and propose some reformulations here and there to simplify the text. As mentioned above, the text has been shortened, removing useless repetitions and explanations. The last (and relatively technical) scaling experiments (in the section about dosage) has been moved into the Supplementary Materials.

2) Overall: Li et al. 2019 showed that DSBs at asymmetric PRDM9 binding sites are less likely to resolve as either COs or NCOs (roughly half as likely for both), suggesting that many asymmetric DSBs likely repair from the sister chromatid at the end of prophase. They also suggest that this should somewhat counteract the rate of hotspot erosion. It would help to acknowledge and clarify this in the text and in the specification of the model (lines 683 and 699).

We now mention the fact that the DSB can be repaired using the sister chromatid in the introduction. We also briefly discuss, at the very end of the article, the consequences (which we think are minor) of ignoring the possibility of sister chromatid repair in the model.

3) Overall: Davies et al., Li et al., and others observed dominance of some PRDM9 alleles over others even without the effects of hotspot erosion, possibly due to inherently different affinity distributions for some alleles over others. For example, the CAST allele was found to be strongly dominant over the Humanized allele in regions of PWD or B6 ancestry, which are not predicted to have experienced erosion of either allele. Does the model account for this erosion-independent dominance?

See above: the model does not explicitly implement intrinsic dominance relations between alleles. We now discuss this point (end of discussion).

4) Abstract and line 22: As you cite later in the manuscript, PRDM9 was first discovered for its role as a meiotic H3K4 methyltransferase essential for fertility (Hayashi et al. 2005), before its role in hybrid sterility was described in 2009.

Well, our point was that the Hst1 locus (which is in fact the PRDM9 gene) was initially discovered by Forejt et al in 1974 for its role in hybrid sterility. However, this point is perhaps not crucial, so we have removed it. This simplifies both the abstract and the introduction.

5) Introduction: It may help a broader readership to have a schematic in the main Figure 1 to illustrate hotspot erosion and highlight the main questions being addressed by the study.

We have added a little figure to that effect.

6) Line 45: It would help to define more broadly what Red Queen means, not only in the context of PRDM9 evolution.

Done, see above, response to AE.

7) Line 98: Be clearer here about what is established mechanism versus speculation/hypothesis.

Done

8) Line 101: please clarify the final sentence of this paragraph.

Done

9) Discussion: It would help to discuss how population structure (violating the panmictic population assumption) is likely to affect standing levels of polymorphism. Some well-labeled speculation about the implications of the model for the specific observed standing variation in different populations of humans/mice would be worthwhile and interesting. For example, you might apply insights from the model to speculatively explain the observed frequency of A/B vs C alleles in different human populations.

We now mention the possibility that population structure could be one of the factors contributing to maintaining a high genetic diversity at the PRDM9 locus. On the other hand, we prefer to refrain from speculating too much about specific alleles, whether in Humans or in the mouse, since any particular history may have been strongly influenced by random drift.

10) Gene/protein nomenclature: There is inconsistent use of PRDM9 nomenclature throughout. PRDM9 (regular, upper case) refers to the protein. PRDM9 (italics, upper case) refers to the gene in humans (or in no species in particular). Prdm9 (italics, lower case) refers to the gene in mice.

Done

Reviewer #2: Uploaded as an attachment

Text of Reviewer #2, together with our response, are given below, after Reviewer 3.

Reviewer #3: Summary

The authors present an interesting and timely treatment of Red Queen dynamics among PRDM9 alleles. The develop a population genetic simulator that explicitly simulates the meiotic features of the system, which is the first time this has been presented (with the exception of the Baker et al. preprint). The authors find that the Red Queen dynamics emerge spontaneously from the simulations, which encode the two countervailing roles of PRDM9, namely the erosion of existing binding sites via biased gene conversion and the creation of new binding sites for new PRDM9 alleles. The authors derive elegant analytical results that capture the explicit parameters of the previously work by Latrille et al., and extend the model to include genetic dosage. They find that the current analytical derivations agree with the previous work, and the simulations agree with the analytical results under the conditions assumed for the simulation.

In addition to showing that the simulation framework produces both monomorphic and polymorphic PRDM9 regimes given reasonable input parameters, the authors also show that running the simulation reproduces results in agreement with experimental results, mostly in mouse, and conclude that their framework is realistic enough to recapitulate known biology. This paper describes how intragenomic Red Queen dynamics arise in a panmictic population, not just a hybrid population, and the developed simulation program can be used in future works.

My comments below are to improve the readability of the figures and text, and to connect the findings to previous work.

Minor comments

1. In Figure 1A, some alleles have two frequency peaks (e.g. the first dark green allele goes up to frequency 0.7, down to 0.2, and back to 0.75). How often is this behavior expected, and does it affect any of the conclusions about a monomorphic regime?

We have added some words about this in the Results section. This kind of phenomenon seems quite rare and does not impact the conclusions of the model.

2. I find it interesting that in the monomorphic simulations, that the mean rate of symmetrical binding and the fertility of older alleles is lower that than of younger alleles (Figure 1, lines 180-181). This is not immediately evident from the figure, so perhaps this could be clarified visually. Does this have any implications on a long time scale, such as either quantity reaching a maximum or the variance decreasing to some minimum?

We do not understand what Reviewer 3 means here.

3. Figure 1D and 1E, line 190: the increase in symmetrical binding (q) and mean fertility (w) at the beginning and end of allele's existence does not appear to be "slight" in all cases. For example, the oldest blue allele has an increase in q of approximately 0.1 at the end, which is

half of its lifetime range of 0.2, presumably because the old blue allele is in a heterozygous state with the new high-affinity yellow allele. If this is accurate, please clarify the text.

We have removed the word 'slight'

4. For the simulations in Figure 3, the some of the analytical predictions deviate from the simulation results, presumably because the parameters violate the assumptions used in the analytical derivation. This is deviation is especially large for fertility in Figure 3F. Providing some guidance on when the analytical approximations should be used, e.g. for a range of values of v, would be helpful.

The range of values of u and v for which the assumptions behind the analytical approximations are met are illustrated on the figure by the green zone. We have added some mention of the threshold used for each of those assumptions in the caption of figure 3.

5. The Introduction would benefit from referencing the model (schematic in Figure 9 and parameters in Table 2) earlier, such as when describing symmetric binding. Also clearly define key terms at first usage, e.g. "symmetrical binding". In addition, the choice of simulation parameters could be justified by citing literature and stating assumptions earlier in the text (some rationale is in the section, "Empirical input parameters", and Table 2).

We have clarified the word 'symmetry' (see above, comments of the AE). As mentioned above, we have added a little figure especially for this part of the introduction where the role of symmetry in facilitating chromosome pairing is explained. We also moved Figure 9 earlier in the text (as new Figure 2), as well as Table 2 (now Table 1), along with a new introductory paragraph at the beginning of the Results section, in which we briefly explain how the model works (so that the Reader does not need to get through the Methods section in order to understand the main points). We believe this should greatly ease understanding the model and the results that are obtained by running it.

Concerning simulation parameters: at that step of the results, the chosen parameter values are not meant to correspond to empirically relevant situations. Instead, the point is to show how the model works, what its different regimes are, etc. We make this point clear in the text.

6. Connection to Latrille et al.: Figure 3A: possibly note the transition from a monomorphic to polymorphic regime in text. This is mentioned in line 323, and the authors could note the value of u at which this occurs at or highlight it in the figure. In addition, it seems that the conclusion in lines 245-246 that in the case of diversity, u is a key factor while v is not, agrees with Latrille et al,. "In this polymorphic regime, diversity at the PRDM9 locus is roughly proportional to Neu (figure 3) and does not strongly depend on the erosion rate vg". If this is the case, perhaps note this.

We now explicitly make the connection with Latrille et al in the text. In our opinion, there is no clear cutoff value for u, underlying the transition from monomorphic to polymorphic. This is more like the usual (and somewhat qualitative) notion that Nu<< 1 or Nu >>1 correspond to mostly fixed / polymorphic regime also in the neutral case.

7. Line 483: the point that haplo-insufficiency causes the eviction of alleles is interesting, especially since it is not observed in Baker's et al. model (which is mentioned in line 604). Perhaps highlight this finding. Line 589: is there any empirical evidence to suggest that the affinity distribution has a moderate variance, as is used in the simulations of this paper?

We now emphasize the point about haplo-insufficiency and eviction more clearly: This low diversity can be explained by the fact that we are in a range of parameters involving an eviction regime due to gene dosage effects (compare Figure 7 A and Figure 6 D,E,F), such that one allele dominates in the population, while all the other alleles are counter-selected (4Ns_0 << -10, Table 1) except during the short phases of allelic replacement. The fact that eviction is caused by gene dosage can be verified by running the model without gene dosage (i.e. c=1, Table 2, line 2), in which case higher levels of diversity are produced. Intermediate levels of gene dosage (c=1.5, Table 2, line 3), on the other hand, give results that are essentially identical to those obtained with a dosage directly proportional to the number of gene copies (c=2).

Note that we come back to this point in much greater detail in the discussion, when comparing our work with that of Baker et al.

Concerning the variance, and more generally the shape, of the affinity distribution: the text in the discussion is already quite clear about this: "Here we use an exponential distribution, which is motivated by results from Chip-seq experiments [20]"). 2)

Visual and typographic

• Figure 1 and 2 aesthetics: label y-axes with variable name (e.g. f, theta). Consider using a color palette that is color-blind friendly. Perhaps use thinner lines to see the trajectories more clearly. Use a thinner line to highlight the red allele.

We added variable name between parentheses and we now use a colorblind palette for all the figures

We cannot plot thinner lines for trajectories (Python doesn't have thinner lines) but we changed with a thinner line for the bold trajectory

• Figure 1 and 2: it is hard to relate the same point across plots given the separation in axes. Could add some dashed lines for clarity (e.g. the inflection point of one curve corresponds to the maxima of another curve).

We have added a grid for a better readability of the graphic

• It is a bit confusing that the Figure 3 simulations results (in blue) are discussed without mentioning the analytical results (orange), which are in the following sub-section. Perhaps add a sentence around line 220 saying as much.

We have added a sentence to that effect:

Figure 5 shows the simulation results (in blue). It also shows the predictions of an analytical approximation (orange), which is described further below.

We changed to a darker green area

- Line 268: Nvd/2h ~= 4Nvg should be Nvd/2h = 4Nvg

Done

- Equation 2: ~= should be =

Done

- Lines 358 and 361: “Figure 4C” should be “Figure 4F”, for the scenario with genetic dosage.

Done

- Table 1 legend: “mean activity theta” should be “mean level of erosion \bar{z}”;

Done

- Figure 9: add more explanation of the visual elements. For example: X is an inactivated site, width of binding sites indicates affinity, Fig 9D wider arrows denote the CO partners. Legend: add space so is “will contribute”.

We added the explanation of the visual elements suggested

We changed “will contribute”

<u>Reviewer #2</u>:

What the authors have done:

The manuscript describes evaluation of a computer simulation model of the evolutionary processes acting on PRDM9-controlled meiotic recombination. This model follows PRML9 alleles and hotspot alleles in thousands of simulated individuals over many thousands of generations. PRDM9 alleles with new hotspot specificities arise by mutation. The presence of the new PRDM9 allele causes the 400 DNA sequences that, by chance, match its specificity to become hotspots. These hotspots are gradually lost as random mutation creates defective hotspot alleles that replace active ones by conversion. Now when a new PRDM9 allele arises

by mutation it invades the population because its fertility is increased by the recombination it causes at its new hotspots. The paper emphasizes the significance of the newly discovered role of PRDM9 in directly promoting synapsis of homologous chromosomes, and incorporates this activity in its model. The authors used their model to investigate the effects of many aspects of PRDM9's action, including mutation rates of both PRDM9 and the hotspot sequences it acts at and PRDM9 dosage effects.

Assessment summary: This is an important piece of work. It has some scientific weaknesses that I think can be easily corrected, and it needs rewriting and figure changes to make the basic results accessible to researchers studying the molecular and cell biology of meiosis as well as to evolutionary theorists.

Major concerns about the science:

1. This work does not show the importance of PRDM9-mediated chromosome synapsis. This would require running 'control' simulations where PDRM9 plays no direct role in synapsis. See Line 186 comment below.

We have done additional control experiments to that effect. We mention those in the Results sections.

2. The initial simulations make a very unrealistic assumption that the concentration of a PRDM9 protein version in the cell does not depend on whether the cell is homozygous or heterozygous for that allele. This does not make biological sense, especially since different hotspots are assigned different affinities for PRDM9. Thinking about this is confounded because the initial model also assumed that PRDM9 was always in excess, making differences in the amounts of protein irrelevant.

Later a more realistic 'Gene dosage' version of the model is introduced, where a meiotic cell homozygous for a particular PRDM9 allele contains twice as much of its version of the protein than a heterozygote does, making symmetrical binding twice as likely and thus dramatically increasing fertility. This dramatically changes the model's predictions. All the effort the reader has put into understanding the complex results in the first part of the paper turns out to have been wasted.

The first part, which does not take into account genetic dosage, is useful from a logical point of view, as it enables us to explain a simpler version of the model and, once the dosage has been introduced, to identify what is due to the dosage effect and what is due to other dosage-independent mechanisms. We added a paragraph at the beginning of the Results section to explain and motivate the successive logical steps of our presentation.

Here's a sketch showing the differing assumptions:

A general note about the writing: I think this article should try much harder to be accessible to both parties interested in meiosis: evolutionary biologists and molecular geneticists/cell

biologists. At present it contains a lot of unexplained and poorly explained evolutionary biology jargon.

See above, our response to AE. We have made our best to correctly introduce and explain all of the necessary terminology, and removed part of the unnecessary jargon.

Many paragraphs are much too long, especially lines 153-185, 423-459 and 672-708. Any paragraphs more than 20 lines long should be re-evaluated.

We have split all of those paragraphs. In fact, they turned out to have relatively natural subparts and thus could be split quite easily.

The authors have been very careless in how they write the name of the PRDM9 gene, of its alleles and of the PRDM9 protein. Between just the Title, the Abstract and the Author summary I found five different combinations of capitalization and italicization, apparently chosen randomly for each mention without regard to whether it was the gene, and allele or the protein. The authors should find out the correct forms and use them consistently.

Done. Sorry for this confusion.

Notes on specific points:

Abstract:

'Red Queen' is evolutionary genetics jargon that won't be understood by cell biologists and many geneticists. Either explain it at the very start (first describe what happens, and only then introduce the term), or don't use it at all in the Abstract. And minimize the use of it in the body of the manuscript too.

We have substantially rewritten the abstract. Concerning the 'Red Queen' terminology, it should be pointed out that this has been the name of the model since its first introduction by Ubeda and Wilkins (who use this terminology in the title of their own article). So, we still use it. We don't have enough space in the abstract to fully explain the origin of this terminology. However, we now say 'the so-called Red Queen model', and then go on explaining in more detail the principle of the model.

In the introduction, we follow the Reviewer's suggestion: first explaining the model, and then we introduce the terminology.

Spell out, early on, that we now know that 'hotspots' promote local crossing-over because they are high-affinity binding sites for PRDM9. This is not clear enough in the current Abstract.

Done

'…hotspots are eroded…': The meaning of 'eroded' is not obvious here; describe what actually happens and explain that the term 'erosion' is used to describe this.

We added this information "[...] leads to the progressive inactivation, hereafter called erosion, of PRDM9 binding sites at the genome scale [...]"

The Abstract ends with: "Finally, calibrating the model based on current empirical knowledge shows there is no need for much more realism in our model to correctly fit the empirical data." It's true that with appropriate choice of parameter values the model's predictions agree with what is seen in real meiosis and populations. But the model makes many simplifying assumptions, and relaxing any of these could dramatically change the outcomes.

Since then, we have realized that the model, under this configuration, was in fact not correctly predicting one important thing that is nevertheless observed in empirical data: strong positive selection on PRDM9. So we have changed the end of this section, as well as our conclusions on the empirical fit.

Author Summary:

Be clear that meiotic recombination occurs in two ways, by random assortment of maternal and paternal homologs into gametes, and by crossing over. You may then, if you choose, say that since the model considers only a single chromosome you will use 'recombination' to mean 'crossing over' in this manuscript.

Again we lack space here to get into those details. Please note that, the way we have formulated things at that point of the summary does not require restricting the scope of 'recombination' to only crossovers.

'instability of the recombination landscape': Most readers will not understand what this is referring to, so give a better explanation of the hotspot paradox.

We now say PRDM9 is crucial in determining the location of recombination points and in promoting chromosome pairing, by binding to specific DNA sequences. This molecular mechanism, however, has paradoxical consequences, characterized by the local destruction of the DNA sequences recognized by PRDM9 proteins, leading to their rapid loss at the level of the population over a short evolutionary time.

Introduction:

Line

10 '..requires to articulate…' Maybe '… requires explicit descriptions of both…'

Done

16 saying '…they cluster…' suggests that a single meiosis would have a cluster of closely spaced crossovers at each hotspot. Maybe say '…crossovers frequently occur at the same positions in independent meioses '.

Done

21: Wait to introduce the 'zinc finger' until line 56.

We now first introduce PRDM9 as a DNA binding protein, and then later on, introduce the zinc-finger domain.

36: The writing will be easier to follow if the terminology is consistent. Instead of 'replacement of 'hot' alleles by allelic motifs…' say 'replacement of 'hot' alleles by alleles…

Done

38: The dBGC abbreviation is unnecessary; don't introduce it.

'mutations that weaken or inactivate hotspots.'

We removed dBGC

42 Again, I don't think 'erosion' is a very helpful term for this process. (It's used 80 times in the manuscript!)

This terminology has been used several times previously, so we prefer to keep it. However, we now clearly define this term the first time it appears in the text.

48 It would be helpful to clarify here that the sequences recognized as hotspot sites by the new PRDM9 allele were already present by chance in the genome.

Done

56 Does the reader need to know that the zinc finger is encoded by a mini-satellite? Why is this significant?

Yes it is very significant – thanks for pointing out this important point. We have added some information about this in the introduction:

Second, the DNA binding domain of PRDM9 is a zinc finger domain, consisting of an array of 7 to 10 zinc-fingers. This domain is encoded by a mini-satellite, which mutates rapidly (Jeffreys et al 2013) by a combination of point mutations and unequal crossing over between the sequence repeats. This allows for the rapid accumulation of new combinations of zinc fingers, and thus of new alleles recognizing different hotspots, providing the necessary mutational input for the Red Queen to run.

62 'Wright-Fisher models'. OK, from here on I'm going to stop suggesting ways to make the manuscript intelligible to meiosis researchers who aren't evolutionary biologists.

In fact, we have realized that the 'Wright-Fisher' terminology does not refer to anything specific, apart from a general model of a reproducing population with non-overlapping generations. So, we have removed this term in the introduction and results section.

68 ‘induced by PRDM9’ is ambiguous. How about ‘seen in PRDM9 heterozygotes with incompatible PRDM9 alleles’?

This is not completely true. The link between the hybrid sterility phenotype and the molecular function is rather indirect and depends both on the PRDM9 genotype and on the genomic background. So, we prefer to stick to our original formulation. The paragraph that immediately follows this sentence explains the whole idea in greater detail.

75 How about ‘In a F1 hybrid, each of the two PRDM9 alleles has eroded its targets in the genome of its parental strain, but not in the other other strain’s genome.’?

Done

77 (They’re not really in trans or in cis, since the PRDM9 allele is only on the homologs of one chromosome.) How about ‘Each PRDM9 allele will therefore tend to bind preferentially to the still active hotspot sites present on the chromosomes inherited from the other parent, but not to the homologous but eroded sites on the chromosome from its own parent.’?

Done

93 Do DSBs often get repaired by pairing with a strand in the sister chromatid? If not I’l leave this out.

Yes they do. We now mention this point and cite Li et al 2019. See above.

96 If the homologs fail to synapse the meiosis will be aborted.

This part has been removed, as a way to shorten the introduction.

103 Huh?

Sorry. We have rephrased: In contrast, in the case where PRDM9 binds only on one of the two homologous loci, a possible DSB would be repaired only later on, either by the homologue as a non CO event or by the sister chromatid.

107-109 Maybe, instead of saying ‘panmictic’, say ‘within a single population’ – this better reinforces the contrast with hybrids formed between members of two separately evolving populations. A diagram would be helpful.

Done

Results

126 Don’t just dump the reader in at the deep end with the model. The Results should start with an overview that briefly describes the components and events of the model and explains how these approximate the components and events of meiosis in a real population. Since the

Methods will be placed at the end of the paper, the authors shouldn't assume that the reader has already read them.

Yes, that is a very important point. We have added a summary of the model at the beginning of the Results section. We have also shifted the former Figure 6 (explaining the simulation model) at that point, as new Figure 2. Of note, we also have shifted the Table introducing the notations at this point of the manuscript (as the new Table 1).

Skipping over to the Methods section:

Is this description correct? (I'm not expecting this to be used to introduce the model – I just want to try to lay out my understanding.)

1. The model simulates one chromosome (2 homologs per individual) in 5000 individuals of a randomly mating population.

Yes

2. Each chromosome has one PRDM9 locus.

Yes

3. Each chromosome has 400 loci that are binding sites for its PRDM9 protein (potential hotspots).

Yes

4. These binding sites have differing affinities for PRDM9 (some are stronger hotspots than others)

Yes

5. The relative positions of these hotspot loci on the chromosome are not specified.

Yes they are: they are explicitly attributed to a newly arisen allele upon its birth.

6. The population is followed over many generations.

Yes

7. Each generation consists of mutation, production of gametes by meiosis and then random fusing of gametes from pairs of individuals to create the next generation of individuals.

Yes

8. When a PRDM9 locus mutates its protein acquires a different hotspot-binding specificity. A new array of 400 hotspot loci is then created for each new PRDM9; these simulate pre-existing DNA sequences that now will act as hotspots for the new PRDM9 protein.

Yes

9. When a hotspot allele mutates it becomes unable to bind its cognate PRDM9 protein (it becomes a cold spot).

Yes

622 Fig. 9 has several problems, some more serious than others:

1. Why do the drawings show the hotspots for PRDM9 alleles that the individual does not have? In Fig. 9A, the first and fourth individuals are homozygous at PDRM9 and thus should have only one type of hotspot (yellow and green respectively). In Fig. 9B, only the third individual has a dark-red PRDM9 allele, so why are the hotspots for this allele shown in all the individuals? And why show the yellow hotspot alleles in this individual, when its only PRDM9 alleles are green and dark red? Similarly, there should be no dark-red hotspots in panels C, D and E. Removing the irrelevant target sites from the drawings will make the representation both simpler and more accurate.

We show all the sites recognized by all PRDM9 alleles present in the population because even if they don't act as recombination sites in an individual carrying alleles that do not recognize them, these sites still exist in this individual and can undergo mutations that may be transmitted to the next generation. Also, if this gamete carrying these mutations is then associated to a homologue carrying an allele recognizing these sites, it is important to know their active/inactive status.

We removed the purple sites in the meiosis step because they have no utility here.

2. The representation of and spacing between sister chromatids in panels B, C, D and E is exactly the same as of homologs in Panel A reinforcing the all-too-common confusion between sisters and homologs. At least draw the sisters as being closer together than the homologs were in Panel A.

Done

3. The Panel B individual whose meiosis is shown in Panels C, D and E should be indicated by a purple rectangle, not a black oval, since Panels C, D and E are enclosed in a purple rectangle.

Done

4. In Panels C and D, binding of PDRM9 to its cognate hotspots should be indicated by filled circles of the appropriate color representing PDRM9 protein (like those representing its gene), not by skinny arrows.

We tried but found it difficult to obtain a figure that was not too cluttered. So, in the end, we reverted to using the arrows.

5. Since Panel D is supposed to represent synapsis of the homologs, put them closer together, at least at the symmetrical site.

Done

6. Are the fat yellow arrows inside the blue rectangle in Panel D supposed to indicate that the homologs are being brought together at the site of the symmetrical binding? It should be possible to find a clearer way to represent this. And what does the skinny-line rectangle below indicate? It has symmetrical binding of PDRM9 but no fat arrows.

The two rectangles represent symmetrical bound sites as shown by the legend. As we consider the simplification that only symmetrical bound sites experiencing DSB can form crossing-over, and that only one crossing-over is performed by meiosis, one of the symmetrically bound site is chosen and is represented by thicker arrows and a thicker blue rectangle with the legend 'CO' above it indicating its signification. We replaced the rectangles by representing the chromatis closer to each other. The two chromatids performing the CO are then shown in panel E as dotted rectangles.

7. The red rectangle in Panel E indicates gene conversion at the top green hotspot

Yes

8. Shouldn't Panel E show the result of the crossover? Instead it shows the boxed chromatid arms still in their original positions.

Yes, Done

9. Panel F is an orphan – I completely overlooked it because it's so far over to the right.

We believe it is still useful to have this last panel, just to make the explanation complete. This panel is referred to in the legend.

10. Don't use similar rectangles to represent different kinds of events and relationships (individual, crossovers, potential crossovers, synapsis, gene conversion).

We now use different types of rectangles for different types of events.

Continuing through the Methods:

629 Maybe have a little plot somewhere showing the distribution of target site affinities at their creation (because I'm not sure what 'an exponential law of parameter y' looks like) and the distribution at the time their cognate PRDM9 allele goes extinct?

We have added a supplementary figure for this - and we refer to Smagulova et al, 2016 for a more direct visualization of the empirical distribution.

639 The two alleles of each new hotspot site are assigned identical affinities. This is reasonable, but with no mutations affecting hotspot affinity, the model can't include conversion of a strong hotspot to a weak one, likely a very important component of hotspot erosion.

Yes, that's an approximation of our current model: at the birth of a new PRDM9 allele, all target sites are active. As a result, erosion can only be the result of new mutations occurring after the birth of the allele. We are now more explicit about this point in the methods.

645 This is reasonable since hotspot alleles newly arisen by mutation would be at a very high risk of being lost by conversion.

Yes indeed. We have added a mention about this point in the methods.

663 There are 400 target loci (hotspot positions), but each is present on four chromatids in the meiotic cell. Is the binding calculation done for each site on each chromatid in turn?

Yes, 400 target loci on 1 chromatide which corresponds to 400x4=1600 sites in total on both homologues, and the binding status of each site is randomly chosen. We have added this information in the text (see just below).

664-672 I do not understand what this analysis is accomplishing.

We now more explicitly say what these equations are used for, which should clarify. We also mention at that point the fact that there are 4 instances of a given binding site:

Using this equation, for each binding site, binding is randomly determined, by drawing a Bernoulli random variable of probability x_i. Of note, at a given target locus, there are four instances of the binding site (one on each of the two sister chromatids for each of the two homologues), and binding is determined independently for each of those instances.

683 'which the model assumes are essential for crossing-over' ?

We changed to "which the model assumes are essential for chromosome pairing"

686: 'on which to hybridize' - replace with 'to base pair with'?

We simply erased this sentence

697 'The chromosomal segments on either side of the chosen site are exchanged' Does 'on either side' mean 'on both sides'? That would not be recombination at all. Should it be 'on one side'?

We have simplified this sentence:

Thus, only one CO is performed per meiosis (and this, in order to model CO interference), and all other DSBs are repaired as NCO events.

702: The hotspot allele at the site of the DSB that became the crossover also gets converted, right? Oh, this is irrelevant if non-dead hotspot alleles are always identical (see line 639 comment above).

Yes. Formally, the CO also has a small gene conversion tract, although in the present case, it cannot lead to actual gene conversion between alternative alleles.

705 …to become a gamete'.

Done

787 'the number of bound sites per allele was set to h = 400'??? What does 'bound sites per allele' mean? I would expect something like 'sites recognized as hotspots by each PDRM9 allele's protein'.

We have changed this accordingly

Results

126 and 128 'Intragenomic Red Queen' and 'Wright Fisher simulation' will only be intelligible to population geneticists.

We have addressed this; see above, response to comments of the AE.

135 The appropriate response on seeing Fig. 1 is "YEOW!!" If you move the figure until after the explanation in the next paragraph it won't be nearly so daunting.

We agree but we were asked to place the figures just after the paragraph where they are first referenced (submission instructions).

Legend to Fig. 1

- 'In all panels, different colors correspond to different PDRM9 alleles that have newly arisen by mutation.'

Done

- Replace 'Successive panels' with 'Each panel'.

Done

- Many events are crowded together in each panel, making it difficult to align events in different panels. Adding faint vertical lines every 5000 generations would help.

Done

139 The paper will be more accessible to cell and molecular biologists if technical terms such as 'monomorphic regime' are only introduced when really needed.

We feel that this point of terminology is quite important. We have made our best to explicitly define those terms when first mentioned in the text.

140 It would be helpful to have a figure explaining the events in the life of a single PRDM9 allele first. It would be most useful if this was a run with a lower μ, so the 'old' allele hangs around longer. See suggestion at line 200 below.

What is now Figure 2 gives most of the relevant information about this. As for showing a figure with an even lower u, we don't think this would add so much (given that the manuscript is already quite long and dense).

How often do we expect a new PRDM9 allele to arise, with μ=5x10-6? N=5000, 2 alleles each, so about one every 500 generations? Yes, that's about what we see.

Yes

The new PRDM9 allele invades because it initially has more active hotspots than the resident allele(s) and thus more successful meioses. But inactive hotspots start to accumulate, initially due to mutation and then also due to conversion of active hotspots. This reduces the meiosis advantage, initially very slowly by mutation and then faster and faster as the probability of conversion rises. But the previous PRDM9 allele can't come back since loss of hotspots is irreversible (though check out the purple one that arises at generation 17,000). So the frequency of the new allele continues to increase until it is fixed or until a new allele arises by mutation. The longer it has persisted the fewer active hotspots remain and the faster it is replaced once a new allele arises.

Yes

Usually, fertility is a property of an organism, not an allele. Is the fertility plotted in Fig. 1E the mean fertility (probability of successful meiosis) of the diploid individuals carrying this allele? Or, as lines 192-3 imply, of all the individuals in the population, regardless of which PRDM9 allele(s) they are carrying? But that can't be right, because the plots show allele-specific fertilities (and allele-specific symmetrical binding probabilities). This certainly needs clarification.

Each color corresponds to an allele. The symmetrical binding and fertility statistics correspond to the mean symmetrical binding and fertility of each individual carrying this allele (the fertility thus depends also of the other alleles with which this allele is associated in heterozygous individuals). We now make this point more explicit in Table 1.

Why does the proportion of active sites rarely fall below 0.6? Are they just not shown once their cognate PRDM9 allele is lost? Even though the old allele still has lots of reasonably active hotspots, it can't produce as many gametes as the new allele and so gradually goes extinct.???

Yes. exactly. At the time of their death, new alleles rarely have a lower activity than 0.6 because the new alleles arriving indeed have more targets and especially more targets of higher affinity which were eroded in the old allele which increases their fertility and thus allows them to be positively selected and invade the population, leaving the old one to disappear little by little. In the case where the PRDM9 locus mutates more rarely, the alleles would have time to erode their targets much more than in the current regime.

141 A reader who has not carefully read the Methods (they're at the end of the paper) won't know what 'activity' means here. It's not a property of each PRDM9 allele, but a biochemical measure of how efficiently a PRDM9 protein can cause crossovers given its current supply of hotspots, right?

This is an instance of unnecessary jargon, which we have therefore removed. we now always explicitly say 'proportion of still active targets', or equivalent expressions.

143 replaced by a newly arisen PRDM9 allele that recognizes a different hotspot sequence motif.

Done

165 'bounding sites'???

Target sites, sorry.

The terminology 'activity' and 'active sites' and 'affinity' creates confusion: 'Activity' is a property of a PRDM9 allele -they all start out with the same activity, but this changes as the allele's target sites change. But being 'active' is a property of a target site (hotspot) – sites start out active and become inactive due to mutation or conversion. Only 'active' sites have 'affinity'; that's a stable property of a given site, indicating how efficiently they bind to PRDM9 protein.

Yes. This is another good reason to drop the use of the terminology 'activity' altogether. Thus, now, and throughout the manuscript, we say ' proportion of active sites for a given allele', or 'proportions of sites for a given allele that are still active', or variations thereof, which makes it clearer that this is not an intrinsic property of an allele.

166-168 The relationship between these two sentences is unclear. Maybe just combine them into one statement.

That was changed according to the suggestion.

169 Delete '…are eroded more rapidly. This last point is also expected, since the sites of high affinity…' and '(the binding probability = cy / 1+cy is higher when the affinity y is higher)'.

This has been simplified a bit: Second, erosion results in a decrease of the mean affinity of the sites that are still active (Figure 3 C). This reflects the fact that sites of high affinity are more often bound by PRDM9, and thus are more often converted by inactive mutant versions.

180 of the affinity of the hotspots for the PRDM9 protein?

Done

181 'all sites of a given allele' is confusing. Instead 'all target sites of a given PRDM9 allele'?

Done

186-189 Well no, the importance of symmetrical binding hasn't been established in the model. How does the model outcome change if the requirement for symmetrical binding is removed? For example, synapsis (and successful meiosis) could instead be made to depend on the number of DSBs in the chromosome.

We have made this control experiment and we mention it in this part of the results section. As it turns out, there is still a turnover of PRDM9 alleles, but erosion is much deeper. Depending on the regime, the turnover is driven either by the replacement of alleles that have lost all of their binding sites (no opportunity anymore for DSBs), or, under higher mutation rates, by a pure mutational turnover at the PRDM9 locus.

192-195 Symmetrical binding probability and fertility 'are defined, for each allele, as a mean over all diploid genotypes segregating in the population at any given time.' This definition makes panels D and E quite misleading. I don't understand how the plots can show what appear to be allele-specific symmetrical binding probabilities and fertilities if this is the case. For example, around generation 16,000 we see a pale green line and a turquoise line, which would seem to imply that the data are specific to the segregating pale green and turquoise PRDM9 alleles.

The thing is, fertility can only be defined at the level of the (diploid) individuals, so it cannot be an intrinsic property of a PRDM9 allele. However, it is still possible to define the mean fertility of an allele, by averaging over all possible genotypes. Owing to the parallel we want to make between fertility and binding symmetry, we think it is better to opt for the same definition for symmetric binding rate. Of note, first, this is the standard way to define the mean fitness of an allele (it is usually the mean fitness of individuals carrying this allele); and second, we are quite clear about this point later in the text:

'The reason of this is that these two summary statistics are defined, for each allele, as a mean over all diploid genotypes carrying this allele segregating in the population at any given time. '

Why does being heterozygous for 'old' and 'new' PDRM9 alleles (with different specificities) increase the likelihood of symmetrical binding? Is frequent symmetrical binding

by two ‘new’ PRDM9 proteins just compensating for the increasingly rare symmetrical binding by two ‘old’ PDRM9 proteins?

Is it just that when the individual is heterozygous there’s only half as much of each type of PRDM9 protein? No, that would explain why the probability of symmetrical binding by the new allele goes up as it becomes more common, but not why the probability of symmetrical binding by the old allele goes up as the new one becomes common.

Yes, exactly. This sentence explains this point:

As a result, when old alleles have declined to a low frequency, they often find themselves in a heterozygous state with new alleles, which restores the rate of symmetrical binding and thus the fertility of the corresponding diploid individual.

This paper really would benefit from a one-cycle figure before the current Fig. 1.

In figures 3, the trajectory of a single allele has been singled out using thicker lines, which already provides a graphical display of the life history of a typical allele.

200 Specifically, this mutation rate is 100 times higher (5 x 10-4) and very unrealistic, since such a mutation rate would generate very many nonfunctional PRDM9 alleles and lead to extinction of the whole population. It would be much more informative to instead show results with a narrower range of μ, say 2 x 10-6 (for an initial explanatory figure), 5x10-6 for what is now Fig. 1 and 2 x 10-5 for what is now Fig. 2.

The simulations in this section are not intended to be empirically realistic, but just to explore the different possible outcomes according to the mutation parameters. Empirical calibrations are done later in the article. We have added a justification of this at the beginning of the results section.

Does the model incorporate any cost to the population of the declining fertility due to hotspot erosion, under either the low or high mutation rate scenarios? If this was in place, and the PRDM9 mutations were modeled to include a reasonable proportion of non-functional recessive alleles, the authors could test a range of mutation rates to see if there is an optimum.

As very often done in population genetics, the model assumes a constant population size and, therefore, we do not incorporate any fertility cost to the population. It would be a good idea to explore this point in a subsequent work, although it would seem in the present case (where the decline in fertility is fairly mild in the end, under empirically reasonable parameter configurations) that this would not lead to important qualitative changes (such as increased risk of extinction of the population). Given the already dense array of ideas and results in the manuscript, we think it is better to leave this out.

209 Perhaps provide a supplementary figure where the panels of Fig. 1 and Fig. 2 are drawn to the same scales.

Done

236 Doesn't this assume that only newly-mutated targets participate in conversion?

Strictly speaking, yes. This relates to the point already mentioned above that, upon the birth of a new PRDM9 allele, all of its targets are assumed to be active (no already segregating inactivating mutations). On the other hand, already segregating mutations would be neutrally segregating, thus at a frequency of the order of 4Nv, and thus the rate of erosion would not be fundamentally different.

There's a big jump in line numbers here because I found the 'scaling' analysis (lines 211-330) hard to follow.

332 In heterozygotes, half of the PRDM9 protein in the cell is from one allele and half is from the other. In the previous version of the model a heterozygote has twice as much total PRDM9 protein as a homozygote, a situation that does not make sense biologically. In the new version of the model the total amount of PRDM9 protein in an individual is the same for heterozygotes and homozygotes.

Yes but the previous model helps us to better understand what is happening in a basic model and then we add dosage and we now can determine what is due to dosage what is not. This point is now motivated at the beginning of the Results section.

345-365 This is a striking result. During the extended regime with a stable high-frequency PRDM9 allele, the proportion of active sites decays to about 0.2 and then stabilizes. Why does it stabilize? And is the bulk of the meiotic recombination being done by the low-frequency alleles? If the model included population fitness would the population just die out?

The stabilization occurs only when the old allele is decreasing in frequency in the population. This pattern is also seen without dosage, and reflects the fact that the rate of erosion is proportional to the frequency of the allele in the population. We mention this point earlier in the text.

It is unlikely that the population would die out in that case, as the selection coefficient associated with new alleles at the time of the turning point (when they become positively selected) is still rather small.

436 The hotspot mutation rate used in the simulations was 500-fold higher,

Yes, because before this part it was not meant to be empirically calibrated – as we now point out at the beginning of the Results section.

437 Explain here (or better, earlier) why the real mutation rate is so high (not point mutations but recombination and indels in the Zn finger). Discuss the nature of the real mutations typically seen and their effect on binding ability and binding specificity.

We give a more explicit mention here of the mutational instability of the minisatellite encoding the zinc-finger domain (a point that we also explained more extensively in the new version of the introduction).

450 'one meiosis per individual' means one meiosis attempt per individual each time it is chosen to attempt meiosis. However, an individual may be chosen several times in one generation. In the new alternative, the chosen individual is allowed several attempts to produce a functional gamete.

Yes.

472 Tell the reader about the typical natural diversity and heterozygosity for PRDM9 alleles earlier (in the Introduction).

We have added a mention about this in the introduction: 'In part because of this high mutation rate, PRDM9 is typically characterized by a high genetic diversity in natural populations.'

The model assumes that new PRDM9 alleles have specificities that don't overlap with the parental allele's specificity,

Yes. This point is mentioned in the methods.

506 "Altogether, we deduce that there is no need for much more realism in our model to correctly fit the empirical data." This is a dangerous statement. The model may make unrealistic assumptions about factors that are critical for determining its outcome, and still produce output that matches available data. For example, the current model assumes that every generation produces enough successful meiosis to create the same number of individuals in the next generation. Relaxing this assumption might show that, when a PRDM9 allele persists for a long time, the population goes extinct because its fertility falls below a critical level.

This has changed in the new version of the manuscript. As it turns out, we have realized that this regime was in fact not so adequate empirically, as it corresponds to a nearly-neutral turnover (which contradicts the empirical data suggesting that the DNA binding domain of PRDM9 is under relatively strong positive selection). Of note, we have added a new experiment in this table (modulating the number of DSBs). And we have reformulated our interpretation of those results. See also the very end of the discussion, which comes back to this empirical calibration experiment.

Discussion

524 No, I don't think the need for the second function of PRDM9 has been shown, because versions that did not have this feature were not tested.

See above: we have added control experiments to show this point.

535-536 ‘hybrid context’ and ‘panmictic context’??? Again, the word ‘panmictic isn’t helpful. Maybe again spell out the situations a bit.

We have removed this sentence (just as a consequence of trying to make our discussion a bit more concise).

545 This description of the ‘gene dosage’ version of the model seems to match what I wrote above (line 332) trying to describe the original version of the model. Since this is the only arrangement that makes biological sense, I don’t understand why all the initial modelling (e.g. Figs 1 and 2) would have been done using a different version. Why waste the reader’s attention on a model version that should be discarded?

Again, we feel it is important to go step by step into the logic of the model (see above).

547 This is because the initial assumption that PRDM9 was not limiting has been removed, right? Baker 2022 cites refs saying hotspots do compete for PRDM9.

No: this is only a consequence of the law of mass action. Competition between targets is a different thing. This distinction between law of mass action / gene dosage, on the one hand, and competition between targets, on the other hand, is developed in the next paragraph of the discussion (comparison with Baker et al). In this subsection about gene dosage, we have added a small mention of the law of mass action: ‘By the law of mass action, everything else being equal, increased dosage results in an increased occupancy of the targets and therefore an increased probability of symmetrical binding and a higher fertility. ‘

As for the refs cited by Baker 2022, it does not seem to us that they imply competition for PRDM9 binding. In particular, Baker et al cite Diagouraga et al 2018, but this article of Diagouraga et al talks about another level of competition: competition between sites already bound by PRDM9 (or already modified at the level of the histones) for recruiting DSBs – this level of competition is included in our model as well.

There would be much to say about these subtle aspects of the chemical equilibrium, and about their impact on the evolutionary dynamics, but we cannot develop on that in the present manuscript, that would be too long and it would require a fair amount of additional work to sort everything out – this is precisely why we say at the end of the section discussing our results against those of Baker et al: ‘More globally, it will be important to unravel the exact roles of affinity distribution, PRDM9 concentration and competition between targets in the evolutionary dynamics.’

623 In Table 2 μ is listed as both a parameter and a variable, with slightly different definitions.

u is the mutation rate per individual, mu=4Nu is the population-scaled mutation rate.

Linkage is not discussed (a search didn’t find the word). Since the model considers only a single chromosome, and the positions of the PRDM9 locus and the hotspots are not specified,

in the Discussion the authors should consider the possible effects of linkage between hotspots and between the PRDM9 locus and the hotspots it acts at.

Well, in fact, we do explicitly specify the position of the PRDM9 locus and the target sites along the chromosome, linkage is implemented in our model. In the end, it does not seem to have much impact . Since the text is already quite long, we did not expand on this point.

Since the hotspots matching each new PRDM9 allele were initially at mutational equilibrium on their genome, new ones are being created by mutation at the same rate that existing ones are destroyed by mutation. It's reasonable for the model to neglect the creation of new ones (they're initially heterozygous so destroyed by gene conversion the first time they're used), but the authors should explain this.

Yes, that's a good point. See above. We now mention this in the methods.
