# Supplementary material for: Bridging the gap between the evolutionary dynamics and the molecular mechanisms of meiosis: A model based exploration of the PRDM9 intra-genomic Red Queen
Source: PLoS Genet. 2024 May 20;20(5):e1011274. doi: 10.1371/journal.pgen.1011274 (PMC11142677; doi:10.1371/journal.pgen.1011274)
Supplement: Attachment — Submitted filename: Review of resubmission.pdf [file pgen.1011274.s012.pdf]

Review of resubmission: PGENETICS-D-23-00420R1 for PLOS Genetics

I still think this is a very interesting and important project. The self-destructive mechanism cells use for meiotic crossing-over has subtle and complex genetic and evolutionary consequences, and the simulation model the authors have developed is a powerful tool for investigating them. Unfortunately, the authors have not done the extra work required to correct the manuscript's two major weaknesses. Without these corrections, the manuscript has little scientific value. It does contain other interesting results, but these are unfortunately obscured by the focus on gene dosage effects.

Weakness 1: Gene dosage error:

In the first half of the Results, the authors present their basic model and analyze its predictions. Unfortunately, this version of the model contains what I consider an important error: cells homozygous at the PRDM9 locus are simulated with only half as much total PRDM9 protein as heterozygous cells. The authors present this error as a deliberate feature: "*Although empirically questionable, this assumption offers a simpler basis for understanding key features of the model and of the resulting evolutionary dynamics.*" But this assumption is not *'empirically questionable'*; it is empirically dead wrong; and I think that it must have originally been made in error rather than for pedagogical reasons.

After extensive analysis of this model's predictions (lines 164-368, Figures 3, 4 and 5), the authors test a model version that correctly simulates equal protein levels in homozygotes and heterozygotes. The authors refer to this as the 'gene dosage model' but below I call it the 'dosage-correct' model; they refer to the other model as the 'no gene dosage' model; I'll call it the 'dosage-error' model.

The dosage-error model does not appear to be intrinsically simpler than the dosage-correct one, but it gives very different predictions (Fig. 6), and the authors spend much of the rest of the Results investigating the causes of the differences. The final section of the Results is devoted to explaining that the failure of the model to give biologically reasonable results with experimentally determined parameter values is due to 'gene dosage' effects, as if their two versions of gene dosage were equally valid. But gene dosage is not a variable in meiotic recombination; it's fixed and its value is 2.

To correct this problem the authors would need to rerun most of the simulations, reanalyze the results and rewrite the Results and the Discussion.

Weakness 2: Inadequate testing of the hypothesis that symmetrical pairing promotes Red Queen dynamics:

The Introduction explains that recent experimental data on PRDM9 activity suggests that crossovers preferentially form at hotspots where PRDM9 has bound to sites on both homologs (symmetric binding). This differs from the standard model, where crossovers are initiated by PRDM9 action at a single hotspot site in one of the four chromatids. The Introduction ends with "*Our specific aim was to test whether the combined effects of biased gene conversion and symmetry provide sufficient ingredients for running an intra-genomic Red Queen, and this, under empirically reasonable parameters values.*"

In the first version of the manuscript, the results did not directly test the role of symmetric binding, since all of the simulations required it. In response to my concern, the authors now present results of control simulations where crossovers form at sites where PDRM9 is only present on one chromatid (lines 217-230). However, they do not give these control results the serious consideration they deserve, instead dismissing them in two sentences as 'unreasonable' and 'for trivial reasons', and the analysis is confounded by the effects of the gene-dosage error.

To correct this problem the authors would need to run the control simulations with the dosage-correct model, having first described the criteria by which the model's need for symmetric binding will be evaluated.

Many major and minor issues:

Abstract

'*whose exact location is determined by the DNA-binding protein PRDM9. To explain these fast evolutionary dynamics…*' This is unnecessarily vague. Hotspots are chromosome positions containing DNA sequences where the protein PRDM9 can bind and cause crossing-over.

'eviction' is an inappropriately active term for the passive displacement of competing alleles.

Author Summary

'is' needed on line 11.

Introduction

Line 5: 'into' instead of 'in'?

Various places: Is 'dynamics' singular or plural?

Line 78: Maybe "… the hybrid sterility seen when mice are heterozygous for certain PRDM9 alleles."

Line 89: '…involved in the sterility phenotype' is unnecessarily vague. How about '…are postulated to cause sterility by reducing recombination'?

Lines 110-112: But erosion is *caused* by conversion acting at hybrid hotspots. This simple description of the model appears to explain the hybrid sterility at the expense of negating the original erosion problem. Is the solution

Legend to Fig. 1. Why are the homolog chromatids more accessible in the symmetrical binding case than in the asymmetrical binding case? Is it just that PRDM9 has removed the histones and made the DNA of both alleles more accessible? Or are the alleles physically closer together because of something the bound PRDM9 has done? is bound PRDM9 causing the axes of the two homologs to move closer together?

Lines 115-116: by 'Red Queen evolutionary dynamics of recombination' do you mean 'erosion of active hotspots and takeover by new PRDM9 alleles'? Why not say that?

Line 134: producing new alleles recognizing different sequence motifs.

Line 134: The reader should be told here how many hotspot sites there are (400 for each PRDM9 specificity).

Line 135: New sites arise with differing binding affinities for their cognate PRDM9 protein (some high affinity, some lower affinity), but the only subsequent mutations the model follows are ones that reduce binding affinity to zero. You now point this out in the Methods, but it should also be considered in the Discussion.

Legend to Fig. 2: *N* diploid ~~diploid~~ individuals, (2*N* chromosomes, pairs of vertical lines).

Legend to Fig. 2: (C-E) Meiosis in a heterozygous individual.

Legend to Fig. 2: What does 'uniformly' mean in 'uniformly at random'? Does the reader need to know this?

Legend to Fig. 2: 'contribute to the next generation' is unnecessarily vague. How about 'which will fuse with another gamete and become part of the next generation'?

Fig. 2: Arrows are not a good representation of PRDM9 protein binding; the reader expects them to represent motion, or to indicate the progression of time. Instead of using ovals to represent the PRDM9 alleles, how about using triangles or diamonds, and then using ovals to represent the PRDM9 proteins?

Line 139: First an individual is randomly chosen to attempt meiosis and reproduce?

Line 141: This affinity is a property of the hotspot locus, not of the PRDM9 protein, right? So maybe say 'the binding affinity of this hotspot site for PRDM9'?

Line 155-156: '…at the binding sites, causing hotspot erosion in the population'.

Line 157: Is 'susceptible' the best word here?

Table 1: How is 'level of erosion of allele' quantified? The fraction of its initial 400 hotspot sites that are still active?

Line 181: Erosion directly due to inactivating mutations is much slower than erosion due to gene conversion, right? Is this spelled out somewhere?

Line 190: Should 'rate of symmetrical binding' be 'frequency of symmetrical binding' or 'probability of symmetrical binding' (as in Table 1)?

Line 191: These equations are in the Methods, right?

Fig. 3: This figure is still quite unnecessarily busy. I don't think any understanding would be compromised by reducing the number of generations shown from 25,000 to, say, 5,000. Or keep A as a top panel, and below expand the first 5,000 generations for all the variables. More generally, almost every figure showing how parameters change over many generations uses a different timescale! (Fig. 3, 25,000; Figs. 4 and S,1 10,000; Fig. 6, 16,000, Figs. 7, 8, and S2, 40,000). Please pick one timescale and use it for all the plots.

Line 207-208: How does 'uniformly at random' differ from just plain random?

Line 214: The mean fertility of an older allele (across all the meiosis it participates in?) is lower.

Lines 214-216: What happens to most of the new PRDM9 alleles that arise when the current allele is still doing well? My previous calculation was wrong; here's the corrected calculation:

> 5×10^{-6} new mutations per PDRM9 allele per generation
>
> x 10,000 alleles in the population
>
> = 5×10^{-2} new PRDM9 mutations/generation
>
> x 700 generations/PRDM9 turnover cycle
>
> = 35 new PRDM9 alleles have arisen during each turnover cycle

Only one of these new alleles succeeded in becoming the dominant allele for the next cycle. What happened to the other 34 alleles (they apparently don't usually persist long enough or achieve high enough levels to be seen in Fig. 3A)? What makes these parameter values give a monomorphic pattern?

The end of each cycle must not be triggered by the origin of a new allele, but by erosion to the point where one of the new alleles can succeed.

What would happen if the mutation rate was lowered, to the point where the original PRDM9 alleles remained dominant after most of its sites had eroded, so that the end of the cycle would be triggered by the availability of a new allele?

Line 221: 'but symmetric binding is not required for chromosome pairing'????

Fig. S2: Because the timescale for Fig S2 is longer than for Fig. 3 (40,000 generations rather than 25,000), it's easy to overlook the longer persistence of each PRDM9 allele in the model that doesn't require symmetrical binding (~1400 generations vs ~700 generations).

Lines 222-223: Why are these levels of erosion considered to be 'unreasonably' high?

Lines 223-225: What makes this a 'trivial' reason? The data in Fig. S2 does not convince me that symmetrical binding by PRDM9 is needed to give biologically reasonable outcomes.

Fig. 4 legend: The allele emphasized by the thick line is far from typical – in fact it's the allele that reached the highest frequency out of the approximately 50,000 alleles arising in this 10,000 generation interval.

Line 267: unclear.

Lines 271-272: The effects of changing the PRDM9 mutation rate are considered twice. First above (lines 245-2632) when the 'polymorphic' condition created by a 100-fold increase in the PRDM9 mutation rate is presented (Fig. 4), and then again in the Scaling Experiments section, where a wide range of rates is examined but only summary data presented Lines 276-285 and Fig. 5A, B and C). But neither investigation discussed the other.

Fig. 5 legend and plots: '…the analytical model verifies the assumptions…' is unclear. From the Methods, I think what's intended is that the assumptions of the model are only true within the green range, so the model's results (orange lines) are invalid outside this area. An easier way to show this for the reader is to only show the model results (orange lines) over that range, with no need for the green shading. I think it's misleading to show the orange lines over the full range of each plot when they are only mathematically valid for the shorter ranges.

Lines 356-365: What do we learn from the model that we didn't already know from the (necessarily more realistic) simulations?

Lines 372-373: This is a rather mealy-mouthed way of admitting that the dosage error was biologically implausible.

Lines 387-388: "…due to gene dosage, homozygotes have a fitness advantage over heterozygotes." Well, in the dosage-correct simulations where symmetric binding is required, homozygotes have an advantage because they produce twice as much of their one PRDM9 variant as each variant produces in a heterozygote, and thus are more likely to achieve simultaneous binding. This advantage should disappear in the control version of the model where symmetric binding is not required.

Lines 387-438: This entire section is a waste of the reader's time. Gene dosage is not a biological variable, and there is no scientific benefit from understanding why the dosage-error model gives different outcomes than the dosage-correct model.

Line 400 and subsequently: I think 'eviction' has been given a special meaning: 'rapidly eliminated from the population while still rare'. If this is the intention, the redefinition needs to be spelled out (the text at Line 400 is not sufficient). Better, just describe what happens, since this new term isn't needed.

Line 461: It would be good to start here with a sentence explaining what an 'empirical calibration of the model' is and why that is desirable.

Notes to Table 2, and Lines 478 and 508: The authors' definition of 'haplo-insufficiency' is correct (difference in fitness of homozygotes and hemizygotes), but there are no hemizygotes in this work, and it's very incorrect to use 'haplo-insufficiency' to describe differences in fitness between homozygous individuals and those heterozygous for two functional alleles. I also can't find any description of how it is calculated. Instead the authors should more clearly describe the phenomenon (and explain how it is calculated), without introducing a new and incorrect term.

Lines 512-513: “*Nevertheless, at least in its current form and under those 512 parameter values, the model does not predict an empirically reasonable regime.*“

Line 551: Which model? Dosage-error or dosage-correct?

763-764: What are the implications of the assumption that the number of DSBs is not dependent on the number of sites where PRDM9 has bound? Does the experimental data cited include genomes where hotspots have undergone significant erosion?

In case you’re interested: The name ‘Red Queen’ is a reference to Lewis Carroll’s Alice in Wonderland, where the Red Queen tells Alice “*Now, here, you see, it takes all the running you can do, to keep in the* same place.”
