# Supplementary material for: Bridging the gap between the evolutionary dynamics and the molecular mechanisms of meiosis: A model based exploration of the PRDM9 intra-genomic Red Queen
Source: PLoS Genet. 2024 May 20;20(5):e1011274. doi: 10.1371/journal.pgen.1011274 (PMC11142677; doi:10.1371/journal.pgen.1011274)
Supplement: Attachment — Submitted filename: GENESTIER_et_al_2023_Response_to_Reviewers.pdf [file pgen.1011274.s013.pdf]

*Dear Dr Genestier,*

*Thank you very much for submitting your Research Article entitled 'Bridging the gap between the evolutionary dynamics and the molecular mechanisms of meiosis: a model based exploration of the PRDM9 intra-genomic Red Queen' to PLOS Genetics.*

*The manuscript was fully evaluated at the editorial level and by an independent peer reviewer. The reviewer appreciated the attention to an important problem, but raised some substantial concerns about the current manuscript. Based on the reviews, we will not be able to accept this version of the manuscript, but we would be willing to consider a much-revised version. We cannot, of course, promise publication at that time.*

*While the revised manuscript thoroughly addresses many of the specific points made by the original reviewers, there remain serious reviewer concerns that a particular, and quite central, aspect of the modelling reduces the impact and usefulness of the results at present: the use and weighting attached to the "no-dosage" model whereby PRDM9 dosage does not impact fertility. Balancing this against the other two reviewer's positive opinions - that the comparison of models brings insights - and even though no-dosage is not used for the whole paper, it still does at present cloud the major insights all reviewers agree this work would otherwise bring. First, there is an issue of how – or if – the model is justified biologically. Second, the reviewer feels much too much weight is given to this model in the manuscript main text and figures, especially if it is seen as a "straw man" hypothesis. It is essential that these points are addressed. There are of course, out of necessity many factors that cannot be taken into account in a model-based approach. None of the reviewers believe that, for example, it is essential to model the impact of numbers/sizes of chromosomes, or multiplicative effects of asynapsis on infertility due to e.g. cytoplasmic bridges between cells, to gain useful insights on evolutionary dynamics from "red queen" like dynamics. However, dosage is more basic than these and has more potential for useful/reasonable modelling.*

→ We provide a more detailed answer below (after points 1 and 2 of AE).

1. *In justifying the "no-dosage" model, the existing approach to say "Although empirically questionable, this assumption offers a simpler basis for understanding key features of the model and of the resulting evolutionary dynamics" was highly ambiguous to the reviewer. Similar is "Note that the parameter values used here are not meant to be empirically relevant". These are insufficiently strong and indeed suggest this model *might* be right or would be correct with appropriate parameter choices. Then, on page 12 the statement "the previous results were obtained with a model assuming the same concentration of the PRDM9 protein product of a given allele in individuals that are either homozygous or heterozygous for this allele" is worrying to the reader, because putting it this way implies the prior work – already read at this point - does not in fact reflect any reasonable biological reality. To be clearer to readers, improved justification of the use of this model needs to be added. First, is there any binding or – perhaps more likely – fertility model in which the "no-*

*dosage" approach would be correct (see point 2), as suggested by "questionable"? OR is it a key boundary case, and e.g. we expect the real world to lie between the no-dosage and completely dosage dependent extremes. As an example suggestion, perhaps this might happen if there are timing issues in meiosis – which is almost certain in reality. A time window for synapsis might allow partial recovery of fertility from a lower level of instantaneous binding in a heterozygote by allowing for synapsis if binding occurs at any point within the time range. This makes synapsis less sensitive to hotspot "heat", and so dosage, but still sensitive to complete hotspot loss - might this allow for a spectrum of heterozygote disadvantage. It might then be appropriate to simply view dosage impact as a parameter, and evaluate the edge cases. Secondly, if not realistic, then given that later comparisons with an obviously unrealistic model are not of immediately clear value, then explain more precisely why are we looking at this model. If it is essential to understand this model, in order to build or understand more realistic models, this should be stated. If this model is more amenable to the theoretical calculations this should similarly be stated – but ultimately, biological relevance of the theoretical calculations should still be justified.*

2. *In terms of weighting, I concur with the reviewer that the main text figures and descriptions currently focus too heavily on the apparently more unrealistic model at the expense of the more realistic models. Currently I believe Figure 3, Figures 4 and 5., and also Figure 6A-C relate only to the no-dosage model. Only in Figure 6D-F is the more realistic model considered. Then Figures 7 and 8 use this model, attempting to fit real-world parameters more precisely. To address the reviewer's main remaining concern, consider reversing the order of discussion to only consider the no-dosage model* ***after*** *the more realistic model. This would allow you to motivate the no-dosage model as a way of allowing the evolution of higher diversity levels, that are often seen in nature - and then allowing discussion of how this feature might occur biologically, at least via reduced dosage. In any case, please ensure that the more realistic model gains at least as much representation in the Figures as the no-dosage model, e.g. by moving most or all of these earlier figures (3-5) to the Supplementary material, or deleting less important ones entirely.*

→ We appreciate the suggestions of the AE as to whether the no-dosage model might after all have some empirical relevance, if but as a boundary case. However, we do not believe this is the case. Given the ever-changing binding specificity of PRDM9, it would require fairly contrived mechanisms for the regulation of expression. So, clearly, the no-dosage model is not empirically relevant. Our phrasing about this in the previous versions of the manuscript might have been inadequate, or at any rate not clear enough (e.g. the use of 'might').

→ Second, the more specific suggestions of the AE (in particular the time window), certainly represent an interesting idea. However, it seems to us that this does not so much modulate the intensity of dosage than its consequences -- in other words, it does not change the factor of 2 at the level of the concentration of each allele. Instead, it plays on the downstream non-linearities, in particular through the value of sigma_0 (the relative difference in fitness between the homozygote

and the heterozygote). In fact, this specific suggestion of the AE is similar to what we have in mind when presenting the very last version of the model, with more DSBs, as an indirect way to account for the progressive introduction of DSBs during a given window of opportunity. Thus, it seems to us that we already deal with this issue: how to make dosage, given that it exists and is almost certainly exactly 2-fold, have less of an impact on fertility -- which is not the same thing as saying that dosage is less extreme than 2-fold.

On the other hand, we still believe that the model without gene dosage is both pedagogically useful and historically relevant.

The pedagogical value of the no-dosage model can be more clearly seen from equation 3 (of the current version), which we reproduce here:

$$d\ln f/dt = -1/2 \, \alpha \, (z - \langle z \rangle) + \sigma_0 \, (f - \langle f \rangle).$$

This equation captures the effects of erosion and dosage separately, as the two terms on the right hand side. The interplay between these two drivers of the Red Queen dynamics (erosion of the targets versus dosage effects) are quite complex and difficult to sort out based on this complete model. Precisely for that reason, we found it difficult to first introduce the results under the model with dosage, and then only introduce the no-dosage control.

On the other hand, the model without dosage has only the first term of the RHS, and thus, by considering it in a first step, we can work out the consequences of this first term, and then only introduce the second one. In the current version of the manuscript, we have tried to express this idea more directly than in the previous version.

Finally, the no-dosage model is also historically relevant. The original model of Ubeda and Wilkins, as well as that explored by Latrille et al, both ignore the consequences of dosage, which clearly shows that the impact of dosage had not been clearly realized thus far in the previous literature. Working out the detailed consequences of gene dosage, by contrasting the two models, is thus a potentially important contribution of our manuscript.

Nevertheless, we agree that, as it stands, our manuscript devotes too much space to the version of the model without gene dosage. Accordingly, we propose to substantially reduce the space devoted to this model, in particular, by removing the entire section about the scaling properties and the analytical approximation under this model. We still start the results section with the no-dosage model, just for making the point about the origin of positive selection on new alleles and introducing the distinction between monomorphic and polymorphic.

In this first section of the manuscript, we are now more explicit about the fundamental reason (pedagogical and conceptual) why we are considering this model first.

Concerning the parameter values used in this first part of the Results, we now write: ‘Note that the parameter values used here are not empirically relevant. Instead, the aim is to illustrate the different regimes produced by the model. An empirical calibration of the model will be presented below.’

Then, we introduce gene dosage (thus, much earlier than in the previous version of the manuscript) and, finally, we work out the scaling and analytical approximations, now under the full model (with dosage), while contrasting it with the no-dosage model. In this section, we have expanded what is now Figure 6, so as to include, not just the diversity, but also the level of erosion, as the summary statistics being monitored. In this figure, we show both models in parallel, given that the contrast between them is a crucial point for explaining the exact impact of gene dosage. It should now be clear that, in this context, the no-dosage model works as a control, or as a contrasting device, a point which we have tried to explain more clearly.

Finally, in the discussion, we re-emphasize the fact that the role of dosage in the Red Queen had not been clearly seen thus far (with the exception of the more recent work of Zach Baker et al, which we duly point out).

A last point: we have changed the parameter settings of the example with which we first introduce gene dosage, so as to match the parameter settings with those of the run shown immediately before it (polymorphic, without dosage).

3. *Existing literature relevant to dosage models. In relevant meiotic cells, the “heat” parameters of your models may in the simplest models be thought of as driven by two other parameters: the “kon” rate at which an individual PRDM9 molecule binds to a single DNA site, and the “koff” rate at which they dissociate from that DNA site. Does the “non-limiting” model of your work correspond to a high value of koff for example, relative to kon? Attempts have been made to estimate PRDM9 behaviour in terms of e.g. koff; see Striedner et al. 2016 for example. Alongside the Paigen lab estimated values for PRDM9 copies per cell, other work from the Forejt lab has suggested that a single DSB at a symmetrically bound site might be enough for a chromosome to synapse (~20Mb of homozygosity being enough for asynapsis to be largely relieved in practice). Please consider citing these prior studies or other relevant ones – and definitely explain how your models fit in with this literature, or how the literature suggests future amendments.*

→ Thanks for the reference to Striedner, which we had missed and is highly relevant. We now cite this work in our discussion. Indeed, the affinity of PRDM9 for its targets can be decomposed in terms of the kon and koff rates. On the other hand, this is only one of the factors determining whether the regime is limiting or non-limiting. The other important factor is the total concentration of PRDM9 in the cell. When the concentration is sufficiently high, we are in a non-limiting regime, otherwise PRDM9 is limiting. The affinity constant will only modulate the concentration at which the transition between those two regimes occurs. As it stands, to our knowledge, there is not much in the literature about the concentration of PRDM9 in the cell. In this respect, as far as we know, the work from Paigen group (we assume this is Baker et al, 2014) does not give an estimate of the number of PRDM9 molecules in the cell. This is qPCR on the targets, which thus gives an estimate of the number of molecules that are bound to DNA. Equating this with the total number of molecules would amount to assuming that PRDM9 is indeed limiting, which is precisely the point about which we would like to get empirical evidence. Concerning the work from the Forejt lab, we cite Gregorova et al, 2018, in the introduction.

4. *Please read the review of the revised manuscript carefully and address all the other specific points raised. In particular, please carefully consider the points made under "Inadequate testing of the hypothesis that symmetrical pairing promotes Red Queen dynamics". I agree with the reviewer that use of the dosage model is important here. I would also highlight that existing literature suggests higher rates of crossover AND noncrossover events at symmetric hotspots. Do you really need to focus on CO events? There are reasons to believe synapsis might not be closely linked to CO (vs NCO) events, but instead more directly to symmetric binding of any DSB.*

→ Concerning the 'inadequate testing': as suggested, we have added the control in the case of the dosage model. Incidentally, the control is a bit complicated to understand, because removing the requirement of symmetry simultaneously suppresses differences between old and young alleles, but also between homo- and heterozygotes. Thus we simultaneously lose positive selection on new alleles and the eviction phenomenon. This again emphasizes the need to introduce those things step by step by first considering the case without dosage (and its control) and then the case with dosage. More globally, we have reformulated the analysis of those controls experiments, so as to make it clear exactly what these controls are useful for – basically, they allow us to establish that the requirement of symmetrical binding provides a selective force that is instrumental in driving the turnover of PRDM9 alleles (see below, response to Reviewer).

→ As for the role of symmetry w.r.t to CO and NCO, based on your feedback, we realize that the following points were perhaps not clear enough: our model fundamentally requires at least 1 DSB in symmetrically bound sites for meiosis to succeed, and then, chooses the (assumed unique) CO among those 'symmetric DSBs'. In hindsight, we realize that this last point is slightly at variance with what we can gather from current literature, which does not explicitly tie this required symmetric

DSB with COs. However, we don't think that choosing the CO among all DSBs would fundamentally change the behavior of the Red Queen, as it would just slightly change the patterns of linkage dissipation (which is in excess anyway).

Perhaps another related point here is the fact that our model does not consider the possibility of repairing DSBs using the sister chromatid, rather than the homologue, as a template. If it did, then it would automatically imply a higher rate of both CO and NCO at symmetrical hot spots. However, as we were already discussing in the previous version, we do not think this would fundamentally change the overall behavior of the Red Queen.

To clarify these issues, we propose to amend our discussion, so as to deal with these two points.

5. *Please correct the following minor issues with the revision not highlighted by the reviewer: please define what w is a function of (i.e. two parameters) in Table 1. Also explain w_hom and w_het and their relationship with w, w*, w bar and the various parameters – at the moment, differing versions of these functions appear across the tables, methods and supplement and it is not always immediately clear how they relate to one another and the other parameters. Also, in Table 1 title, fix the typo "Descritption".*

→ We have clarified this point. One main reason for these different versions of the same quantity (such as w) is that there is a difference in the formalism required for the simulations (in which those variables are generally seen as functions of absolute time, indexed by a specific allele, e.g. w^hom_{i,t}, or two alleles, w^het_{i,j,t}), whereas in the analytical approximation, the same quantities are recast as functions of the intrinsic age of the allele(s) (e.g.  w^hom(z), w^het(z1, z2)).

→ As a way to clarify this, we have pushed all equations expressed in terms of the z-formalism in the supplementary information (where we have added a paragraph to clarify the differences in notation that occur between this supplementary material and the main text). We have also added some details in the methods, as to how exactly we calculate those summary statistics (such as q, w, sigma, s0).

Reviewer's Responses to Questions

**Comments to the Authors:**
**Please note here if the review is uploaded as an attachment.**

*Reviewer #2: Uploaded as an attachment*

*Review of resubmission: PGENETICS-D-23-00420R1 for PLOS Genetics*

*I still think this is a very interesting and important project. The self-destructive mechanism cells use for meiotic crossing-over has subtle and complex genetic and evolutionary consequences, and the simulation model the authors have developed is a powerful tool for investigating them. Unfortunately, the authors have not done the extra work required to correct the manuscript's two major weaknesses. Without these corrections, the manuscript has little scientific value. It does contain other interesting results, but these are unfortunately obscured by the focus on gene dosage effects.*

*Weakness 1: Gene dosage error:*

*In the first half of the Results, the authors present their basic model and analyze its predictions. Unfortunately, this version of the model contains what I consider an important error: cells homozygous at the PRDM9 locus are simulated with only half as much total PRDM9 protein as heterozygous cells. The authors present this error as a deliberate feature: "Although empirically questionable, this assumption offers a simpler basis for understanding key features of the model and of the resulting evolutionary dynamics." But this assumption is not 'empirically questionable'; it is empirically dead wrong; and I think that it must have originally been made in error rather than for pedagogical reasons.*

*After extensive analysis of this model's predictions (lines 164-368, Figures 3, 4 and 5), the authors test a model version that correctly simulates equal protein levels in homozygotes and heterozygotes. The authors refer to this as the 'gene dosage model' but below I call it the 'dosage-correct' model; they refer to the other model as the 'no gene dosage' model; I'll call it the 'dosage-error' model.*

*The dosage-error model does not appear to be intrinsically simpler than the dosage-correct one, but it gives very different predictions (Fig. 6), and the authors spend much of the rest of the Results investigating the causes of the differences. The final section of the Results is devoted to explaining that the failure of the model to give biologically reasonable results with experimentally determined parameter values is due to 'gene dosage' effects, as if their two versions of gene dosage were equally valid. But gene dosage is not a variable in meiotic recombination; it's fixed and its value is 2. To correct this problem the authors would need to rerun most of the simulations, reanalyze the results and rewrite the Results and the Discussion.*

See above, response to AE. We agree that our previous version of the manuscript (1) was perhaps not clear enough on the fact that the no-dosage model is empirically inadequate; and (2) was devoting too much space to the no-dosage model. Still, we would like to emphasize that the no-dosage model is intrinsically simpler than the model including dosage (see above, equation and response to AE). This is certainly the most fundamental reason for introducing it, as a first step towards a more expanded model that accounts for all aspects.

*Weakness 2: Inadequate testing of the hypothesis that symmetrical pairing promotes Red Queen dynamics: The Introduction explains that recent experimental data on PRDM9 activity suggests that crossovers preferentially form at hotspots where PRDM9 has bound to sites on both homologs (symmetric binding). This differs from the standard model, where crossovers are initiated by PRDM9 action at a single hotspot site in one of the four chromatids. The Introduction ends with "Our specific aim was to test whether the combined effects of biased gene conversion and*

*symmetry provide sufficient ingredients for running an intra-genomic Red Queen, and this, under empirically reasonable parameters values." In the first version of the manuscript, the results did not directly test the role of symmetric binding, since all of the simulations required it. In response to my concern, the authors now present results of control simulations where crossovers form at sites where PDRM9 is only present on one chroma$d (lines 217-230). However, they do not give these control results the serious consideration they deserve, instead dismissing them in two sentences as 'unreasonable' and 'for trivial reasons', and the analysis is confounded by the effects of the gene-dosage error. To correct this problem the authors would need to run the control simulations with the dosage-correct model, having first described the criteria by which the model's need for symmetric binding will be evaluated.*

→ We have now introduced a control directly in the context of the dosage model. We still present the control experiments in the case of the no-dosage model (both for the monomorphic and the polymorphic regimes). The reason for this is that, again, in the context of the dosage model, the control turns out to have a rather complex behavior, since deactivating the symmetry requirement plays simultaneously on the impact of erosion and the impact of gene dosage. So, it is difficult to make sense of this control without any further preliminary experiment in the absence of dosage.

→ Second, although our initial perspective was to test the hypothesis that erosion + symmetry would provide sufficient ingredients for running the Red Queen, in the end, things turn out to be a bit more subtle, since, without the requirement of symmetry, there is still a turnover, but either a neutral one, or one that is due to a lack of targets for DSB induction. In the current version, we propose to reformulate the overall objective of our analysis in a way that makes more sense with respect to this shift in perspective.

First, we propose to reformulate the aim in the introduction:

'Our specific aim was to investigate whether the combined effects of biased gene conversion and symmetry provide sufficient ingredients for running an intra-genomic Red Queen able to explain current empirical observations.'

Second, we have rephrased our description of the controls, in particular removing qualifiers such as 'unreasonably' and 'trivial'. Instead, we emphasize what we think is the fundamental point: that the requirement of symmetrical binding provides a selective force that is instrumental in driving the turnover of PRDM9 alleles.

*Many major and minor issues:*

*Abstract*

'*whose exact location is determined by the DNA-binding protein PRDM9. To explain these fast evolutionary dynamics…' This is unnecessarily vague. Hotspots are chromosome positions containing DNA sequences where the protein PRDM9 can bind and cause crossing-over.*

→ Done

'*eviction' is an inappropriately active term for the passive displacement of competing alleles.*

→ It is not totally clear to us what should be considered active / passive in the present case. The Reviewer is using the word 'competition', but then, eviction is simply the outcome of this competition between alleles. So, we propose to maintain our terminology. On the other hand, we have defined it more clearly.

*Author Summary*

'*is' needed on line 11.*

→ Not clear to us where this would be needed.

*Introduction*

*Line 5: 'into' instead of 'in'?*

→ Done

*Various places: Is 'dynamics' singular or plural?*

→ We now mostly use dynamic, except if there is an explicit reference to multiple possible dynamics.

*Line 78: Maybe "… the hybrid sterility seen when mice are heterozygous for certain PRDM9 alleles."*

→ Done

*Line 89: '…involved in the sterility phenotype' is unnecessarily vague. How about '…are postulated to cause sterility by reducing recombination'?*

→ we propose: 'are suspected to cause hybrid sterility'. It seems to us that it would be inadequate to say 'by reducing recombination', as it is not quite the issue.

*Lines 110-112: But erosion is caused by conversion acting at hybrid hotspots. This simple description of the model appears to explain the hybrid sterility at the expense of negating the original erosion problem. Is the solution*

→ Not really. It would negate erosion if repair was systematically with the sister, but this is empirically not the case.

*Legend to Fig. 1. Why are the homolog chromatids more accessible in the symmetrical binding case than in the asymmetrical binding case? Is it just that PRDM9 has removed the histones and made the DNA of both alleles more accessible? Or are the alleles physically closer together because of something the bound PRDM9 has done? is bound PRDM9 causing the axes of the two homologs to move closer together?*

→ This point is not totally clear in the current literature. One can speculate that, by restricting homology search in the vicinity of the chromosomal axes reduces the size of the search space, which, statistically, should increase the probability of success of chromosome pairing. How exactly

this is implemented is not clear. We propose to add a mention about this, both in the legend of figure 1, and in the introduction, where we already discuss this point.

*Lines 115-116: by ‘Red Queen evolutionary dynamics of recombination’ do you mean ‘erosion of active hotspots and takeover by new PRDM9 alleles’? Why not say that?*

→ The Red Queen model was introduced earlier, and essentially refers to this erosion/takeover process, so we think we can directly use this terminology at this point of the introduction.

More generally, we need those words: ‘Red Queen’, ‘mono/polymorphic’, ‘eviction’. These are key words by which we try to capture the main properties of an otherwise complex system (which we then try to characterize, both analytically and empirically). We have tried to do our best to define them clearly when first introduced.

*Line 134: producing new alleles recognizing different sequence motifs.*

→ Done

*Line 134: The reader should be told here how many hotspot sites there are (400 for each PRDM9 specificity).*

→ The number of hotspot is 400 in the first part due to computational and time constraints (and is now the number specified in table 1), and 800 in the second part only for the empirical calibrations and thus only specified in this part.

*Line 135: New sites arise with differing binding affinities for their cognate PRDM9 protein (some high affinity, some lower affinity), but the only subsequent mutations the model follows are ones that reduce binding affinity to zero. You now point this out in the Methods, but it should also be considered in the Discussion.*

→ We have added a point about this in the discussion

*Legend to Fig. 2: N diploid diploid individuals, (2N chromosomes, pairs of vertical lines).*

→ Done

*Legend to Fig. 2: (C-E) Meiosis in a heterozygous individual.*

→ Done

*Legend to Fig. 2: What does ‘uniformly’ mean in ‘uniformly at random’? Does the reader need to know this?*

→ this is a standard terminology. We now make it more explicit: ‘one symmetrical DSB is chosen uniformly at random (i.e. all eligible sites have the same probability of being chosen)’.

*Legend to Fig. 2: ‘contribute to the next generation’ is unnecessarily vague. How about ‘which will fuse with another gamete and become part of the next generation’?*

→ Done

*Fig. 2: Arrows are not a good representation of PRDM9 protein binding; the reader expects them to represent motion, or to indicate the progression of time. Instead of using ovals to represent the PRDM9 alleles, how about using triangles or diamonds, and then using ovals to represent the PRDM9 proteins?*

→ We changed the arrows into circles

*Line 139: First an individual is randomly chosen to attempt meiosis and reproduce?*

→ Done

*Line 141: This affinity is a property of the hotspot locus, not of the PRDM9 protein, right? So maybe say 'the binding affinity of this hotspot site for PRDM9'?*

→ Done

*Line 155-156: '…at the binding sites, causing hotspot erosion in the population'.*

→ Done

*Line 157: Is 'susceptible' the best word here?*

→ We have changed to: 'The main question is then to what extent these two aspects of the molecular mechanism WILL influence the evolutionary dynamics.'

*Table 1: How is 'level of erosion of allele' quantified? The fraction of its initial 400 hotspot sites that are still active?*

→ The level of erosion is the proportion of sites that has been inactivated for a given allele. We added an explanation in Table 1

*Line 181: Erosion directly due to inactivating mutations is much slower than erosion due to gene conversion, right? Is this spelled out somewhere?*

→ well, the intended meaning is: by inactivating mutations that are then driven to fixation by biased gene conversion.

*Line 190: Should 'rate of symmetrical binding' be 'frequency of symmetrical binding' or 'probability of symmetrical binding' (as in Table 1)?*

→ Done

*Line 191: These equations are in the Methods, right?*

→ yes

*Fig. 3: This figure is still quite unnecessarily busy. I don't think any understanding would be compromised by reducing the number of generations shown from 25,000 to, say, 5,000. Or keep A as a top panel, and below expand the first 5,000 generations for all the variables. More generally, almost every figure showing how parameters change over many generations uses a different*

*timescale! (Fig. 3, 25,000; Figs. 4 and S,1 10,000; Fig. 6, 16,000, Figs. 7, 8, and S2, 40,000). Please pick one timescale and use it for all the plots.*

→ Done

*Line 207-208: How does 'uniformly at random' differ from just plain random?*

→ in itself, random does not mean much, if we don't specify the distribution from which random sampling is implemented. Here, we maintain that it is important to specify that all sites have the same probability of being chosen.

*Line 214: The mean fertility of an older allele (across all the meiosis it participates in?) is lower.*

→ Done

*Lines 214-216: What happens to most of the new PRDM9 alleles that arise when the current allele is still doing well? My previous calculation was wrong; here's the corrected calculation:*

*5 x 10-6 new mutations per PDRM9 allele per generation*

*x 10,000 alleles in the population*

*= 5 x 10-2 new PRDM9 mutations/generation*

*x 700 generations/PRDM9 turnover cycle*

*= 35 new PRDM9 alleles have arisen during each turnover cycle*

*Only one of these new alleles succeeded in becoming the dominant allele for the next cycle. What happened to the other 34 alleles (they apparently don't usually persist long enough or achieve high enough levels to be seen in Fig. 3A)? What makes these parameter values give a monomorphic pattern?*

*The end of each cycle must not be triggered by the origin of a new allele, but by erosion to the point where one of the new alleles can succeed.*

*What would happen if the mutation rate was lowered, to the point where the original PRDM9 alleles remained dominant after most of its sites had eroded, so that the end of the cycle would be triggered by the availability of a new allele?*

→ The end of a cycle is not triggered in a deterministic manner. This relates to a more general fact of evolutionary genetics, that the probability of invasion of a beneficial mutation (which is of the order of 2s), is generally much less than 1, so it is not surprising that the majority of new alleles never make it, even if they could have. In the present case, as the currently dominating allele becomes older, s is increasing, and therefore the rate of invasion is increasing through the cycle, but the time of the next invasion is still random.

*Line 221: 'but symmetric binding is not required for chromosome pairing'????*

→ We changed to : "this corresponds to a model where PRDM9 is required for the formation of DSBs, but symmetric binding is not required for chromosome pairing"

*Fig. S2: Because the timescale for Fig S2 is longer than for Fig. 3 (40,000 generations rather than 25,000), it's easy to overlook the longer persistence of each PRDM9 allele in the model that doesn't require symmetrical binding (~1400 generations vs ~700 generations).*

→ We changed the time scale, now it's the same across all figures.

*Lines 222-223: Why are these levels of erosion considered to be 'unreasonably' high?*

→ It is high compared with empirically observed levels (which are more of the order ot 20%). However, at that point of the text, we are not yet dealing with empirical observations, so perhaps this 'unreasonably' is not warranted, and we have removed it. What matters at that point of the argument is that it is higher than when symmetrical binding is required – which then shows that differences in fertility mediated by the requirement of symmetrical binding does indeed provide the selective force promoting the turnover of alleles.

*Lines 223-225: What makes this a 'trivial' reason? The data in Fig. S2 does not convince me that symmetrical binding by PRDM9 is needed to give biologically reasonable outcomes.*

→ We have removed the word 'trivial'.

*Fig. 4 legend: The allele emphasized by the thick line is far from typical – in fact it's the allele that reached the highest frequency out of the approximately 50,000 alleles arising in this 10,000 generation interval.*

→ We changed it

*Line 267: unclear.*

→ we have changed this part.

*Lines 271-272: The effects of changing the PRDM9 mutation rate are considered twice. First above (lines 245- 2632) when the 'polymorphic' condition created by a 100-fold increase in the PRDM9 mutation rate is presented (Fig. 4), and then again in the Scaling Experiments section, where a wide range of rates is examined but only summary data presented Lines 276-285 and Fig. 5A, B and C). But neither investigation discussed the other.*

→ the 100-fold increase is there to contrast the monomorphic versus polymorphic regimes, but it is far from giving a more general picture of the quantitative impact of u on the equilibrium regime. Anyway, this has changed, since we now consider the scaling experiments directly in the context of the model with dosage.

*Fig. 5 legend and plots: '…the analytical model verifies the assumptions…' is unclear. From the Methods, I think what's intended is that the assumptions of the model are only true within the green range, so the model's results (orange lines) are invalid outside this area. An easier way to show this for the reader is to only show the model results (orange lines) over that range, with no need for the green shading. I think it's misleading to show the orange lines over the full range of each plot when they are only mathematically valid for the shorter ranges.*

→ this has moved.

*Lines 356-365: What do we learn from the model that we didn't already know from the (necessarily more realistic) simulations?*

→ we advocate the use of math as a way to capture more precisely the logic behind the process (see Servedio et al, 2014, Not Just a Theory—The Utility of Mathematical Models in Evolutionary Biology. PLOS Biology. 2014;12(12):e1002017.

*Lines 372-373: This is a rather mealy-mouthed way of admitting that the dosage error was biologically implausible.*

→ see above: we have tried to be much clearer about the fact that this model is indeed implausible, and about the fundamental reason why the no-dosage model is being considered in our manuscript.

*Lines 387-388: "…due to gene dosage, homozygotes have a fitness advantage over heterozygotes." Well, in the dosage-correct simulations where symmetric binding is required, homozygotes have an advantage because they produce twice as much of their one PRDM9 variant as each variant produces in a heterozygote, and thus are more likely to achieve simultaneous binding. This advantage should disappear in the control version of the model where symmetric binding is not required.*

→ yes it is true, we added a control simulation (see above).

*Lines 387-438: This entire section is a waste of the reader's time. Gene dosage is not a biological variable, and there is no scientific benefit from understanding why the dosage-error model gives different outcomes than the dosage-correct model.*

→ see above. It is quite important to understand the eviction phenomenon, which has not been characterized thus far.

*Line 400 and subsequently: I think 'eviction' has been given a special meaning: 'rapidly eliminated from the population while still rare'. If this is the intention, the redefinition needs to be spelled out (the text at Line 400 is not sufficient). Better, just describe what happens, since this new term isn't needed.*

→ We think that this phenomenon by which alleles that are intrinsically equivalent compete until only one of them wins is central: this is precisely one of the most important consequences of gene dosage – the fundamental reason why we need to proceed step by step, first without and then with dosage, so as to show its existence and characterize the conditions under which it takes place. So, we think it is important to give it a name, and 'eviction' seems to us to be a good name for this. We now define it explicitly.

*Line 461: It would be good to start here with a sentence explaining what an 'empirical calibration of the model' is and why that is desirable.*

→ We have added: 'Finally an empirical calibration of the model was attempted. The idea of this calibration is to try to match the parameters of the model to known empirical values – here, based on current knowledge in mammals and, more specifically, in the mouse, so as to see whether the model is able to at least roughly reproduce key empirical observations, such as the typical erosion levels or genetic diversity at the PRDM9 locus observed in species such as the mouse.'

*Notes to Table 2, and Lines 478 and 508: The authors' definition of 'haplo-insufficiency' is correct (difference in fitness of homozygotes and hemizygotes), but there are no hemizygotes in this work, and it's very incorrect to use 'haplo-insufficiency' to describe differences in fitness between homozygous individuals and those heterozygous for two functional alleles. I also can't find any description of how it is calculated. Instead the authors should more clearly describe the phenomenon (and explain how it is calculated), without introducing a new and incorrect term.*

→ yes, this is an important point that requires clarification: when introducing sigma_0, we now add a mention that the fitness of a heterozygote for two alleles of the same age is the same as that of a hemizygote.

*Lines 512-513: "Nevertheless, at least in its current form and under those parameter values, the model does not predict an empirically reasonable regime."*

→ yes, we maintain.

*Line 551: Which model? Dosage-error or dosage-correct?*

→ with dosage. this should now be clear, in the new version of the manuscript

*763-764: What are the implications of the assumption that the number of DSBs is not dependent on the number of sites where PRDM9 has bound? Does the experimental data cited include genomes where hotspots have undergone significant erosion?*

→ The point concerning the number of DSBs is addressed in the methods: 'This procedure aims to model the regulation of 763 the total number of DSBs through the genome, which in mammals seems to be 764 independent from PRDM9 binding [1,14]'. It is not clear to us what the second question is meant to refer to.

*In case you're interested: The name 'Red Queen' is a reference to Lewis Carroll's Alice in Wonderland, where the Red Queen tells Alice "Now, here, you see, it takes all the running you can do, to keep in the same place."*
